# Prior distributions for variance parameters in a sparse‐event meta‐analysis of a few small trials

**DOI:** 10.1002/pst.2053

**Published:** 2020-08-06

**Authors:** Konstantinos Pateras, Stavros Nikolakopoulos, Kit C. B. Roes

**Affiliations:** ^1^ Department of Biostatistics and Research Support, Julius Center for Health Sciences and Primary Care University medical center Utrecht Utrecht The Netherlands; ^2^ Department of Health Evidence, Section Biostatistics Radboud University Medical Centre Nijmegen The Netherlands

**Keywords:** Bayesian, heterogeneity, meta‐analysis, rare diseases, rare events

## Abstract

In rare diseases, typically only a small number of patients are available for a randomized clinical trial. Nevertheless, it is not uncommon that more than one study is performed to evaluate a (new) treatment. Scarcity of available evidence makes it particularly valuable to pool the data in a meta‐analysis. When the primary outcome is binary, the small sample sizes increase the chance of observing zero events. The frequentist random‐effects model is known to induce bias and to result in improper interval estimation of the overall treatment effect in a meta‐analysis with zero events. Bayesian hierarchical modeling could be a promising alternative. Bayesian models are known for being sensitive to the choice of prior distributions for between‐study variance (heterogeneity) in sparse settings. In a rare disease setting, only limited data will be available to base the prior on, therefore, robustness of estimation is desirable. We performed an extensive and diverse simulation study, aiming to provide practitioners with advice on the choice of a sufficiently robust prior distribution shape for the heterogeneity parameter. Our results show that priors that place some concentrated mass on small *τ* values but do not restrict the density for example, the *Uniform*(−10, 10) heterogeneity prior on the log(*τ* ^2^) scale, show robust 95% coverage combined with less overestimation of the overall treatment effect, across varying degrees of heterogeneity. We illustrate the results with meta‐analyzes of a few small trials.

**Abbreviations**
RCTs: randomized Clinical Trials
MA: meta‐analysis
logOR: log odds ratio
CrI: credible interval

## 1 INTRODUCTION

To reach firm conclusions, randomized controlled trials (RCTs) commonly require large enough sample sizes, but this is not always feasible for (very) rare diseases^1^ in which the limited patient population leads naturally to small RCTs.^2^ In RCTs, dichotomous outcomes are common as they facilitate straightforward clinical interpretation for both efficacy and safety. When combined with small sample sizes and low to moderate event rates, such outcomes lead to a large probability of observing zero events on one or more trial arms.

Even in rare diseases usually more than one trial is available for evaluating a (new) treatment.^3^, ^4^ The small sample sizes make it particularly valuable to pool the data in a meta‐analysis (MA). To synthesize available RCTs, the standard random‐effects MA model is usually applied, also known as the normal‐normal hierarchical model.

When zero events are observed, a complication arises for commonly employed frequentist MA methods. Continuity corrections are needed, usually through adding a constant number to the zero cells. These corrections may affect the study‐specific treatment effect estimates and inflate their variances.^5^ Kuss evaluated likelihood‐based MA methods,^6^ which incorporate information from trials with zero events in one or both treatment arms without the use of such corrections and showed that these performed adequately in settings with non‐small samples and a sufficient number of RCTs in the MA. In a similar setting, either variations on the type of treatment effect measure or the use of the Mantel‐Haenszel method has been suggested in previous simulation studies.^5^, ^6^, ^7^, ^8^

Bayesian MA methods were shown to perform more robustly in MA with only a few small trials.^9^, ^10^, ^11^, ^12^ When synthesizing conveniently large trials, the choice of prior distributions does not impact inference considerably.^13^, ^14^, ^15^, ^16^, ^17^ On the contrary, when pooling a few small trials, only a small number of observations contribute to the model likelihood, therefore, inference becomes prior driven.^18^ For the normal‐normal hierarchical model, a reference prior was suggested that has the ability to maximize the data impact on inference.^11^ Under a normal‐normal hierarchical model, the use of priors that cover plausible heterogeneity (*τ*) ranges has been advocated for a Bayesian MA of a few trials.^10^, ^19^, ^20^ Such priors may not behave similarly when there are zero events in one or both arms, and specific choices of prior shapes may be preferable; that is, according to the way they distribute prior mass across *τ* − values. The normal‐normal hierarchical model has been shown to perform poorly in the presence of zero events in a meta‐analysis of rare diseases.^21^ The use of different distributional model assumptions such as the binomial‐normal hierarchical model may be preferable as (a) it avoids the need for continuity corrections, (b) it directly models the events through a logit link function and (c) it can impose dissimilar baseline effects.

The focus of this paper is to investigate the impact of alternative heterogeneity priors on the (interval) estimation of the overall treatment effect and to provide suggestions for a robust Bayesian MA of a few small sparse‐event trials. Robust priors should retain sensible and predictable operational characteristics throughout a range of unknown parameter values. The paper is organized as follows. In Section 2 we describe a basic Bayesian MA hierarchical model, along with different types of heterogeneity priors. Section 3 presents two motivating examples and their analysis. In Sections 4 and 5 we describe a simulation study that evaluates the selection of priors. In Section 6 we revisit the examples. Finally, in Section 7, we summarize the main findings, while the paper ends with a discussion, as well as recommendations for practitioners.

## 2 BAYESIAN INFERENCE IN META‐ANALYSIS

### 2.1 Bayesian hierarchical model for meta‐analysis

We consider a set of *k* two‐armed RCTs with a binary outcome; patients are randomized over two groups: treatment (T) and control (C) resulting in a 2 × 2 table (Table 1).

**TABLE 1:** Two‐way table notating the *i*th trial of a meta‐analysis

	Treatment	Control	Total
Events	*r*_*iC*_	*r*_*iT*_	*m*_*i*_
Non Events	*n*_*iC*_ − *r*_*iC*_	*n*_*iT*_ − *r*_*iT*_	*N*_*i*_ − *m*_*i*_
Total	*n*_*iC*_	*n*_*iT*_	*N*_*i*_

In each trial *i* ∈ (1, 2, …, *k*) and treatment group *j* ∈{*C*, *T*}, the number of events is modeled to follow a binomial distribution *r*_*ij*_ ∼ *Binomial*(*π*_*ij*_, *n*_*ij*_). By *π*_*ij*_ we denote the probability of an event and by *n*_*ij*_ the number of subjects of treatment arm *j* of trial *i*.^22^ Under a random‐effects assumption, a commonly‐used Bayesian two‐level binomial‐normal hierarchical model^23^, ^24^ can be written, using the control group as reference, as follows:

(1) $$\begin{array}{l} r_{\mathrm{ij}}∼\text{Binomial}\left(\pi_{\mathrm{ij}},n_{\mathrm{ij}}\right)\\ \log\mathrm{it}\left(\pi_{\mathrm{iT}}\right)=\mu_{i}+0.5*\delta_{i}\\ \log\mathrm{it}\left(\pi_{\mathrm{iC}}\right)=\mu_{i}-0.5*\delta_{i} \end{array}$$

where *δ*_*i*_ ∼ *N*(*δ*, *τ* ^2^), so that *τ* ^2^ denotes the between‐study variance and *δ*_*i*_ denotes the study‐specific effects of treatment vs control on the log odds ratio (logOR) scale.

We assume a fixed weakly diffuse normal prior on the overall treatment effect *δ* ∼ *N*(0, 100) throughout and a diffuse normal prior on *μ*_*i*_ ∼ *N*(*μ*_0_, 100) centered around $\mu_{0}=\sum_{i=1}^{k}\mu_{i}/k$_._ ^25^ In comparison to another common choice of hyper‐parameter variance value *δ* ∼ *N*(0, 1000), we lowered the assumed prior variance to produce more stable inferences.^26^ The chosen prior on *δ* has a 95% range of (−19.6, 19.6) in the logOR scale. The heterogeneity parameter can be modeled through alternative prior distributions so that for a transformation of *τ*, *g*(*τ*) ∼ *f*(.), where *g*(*τ*) denotes a transformation of *τ* and *f*(.) denotes a probability density function.

### 2.2 Priors on heterogeneity

**FIGURE 1:**
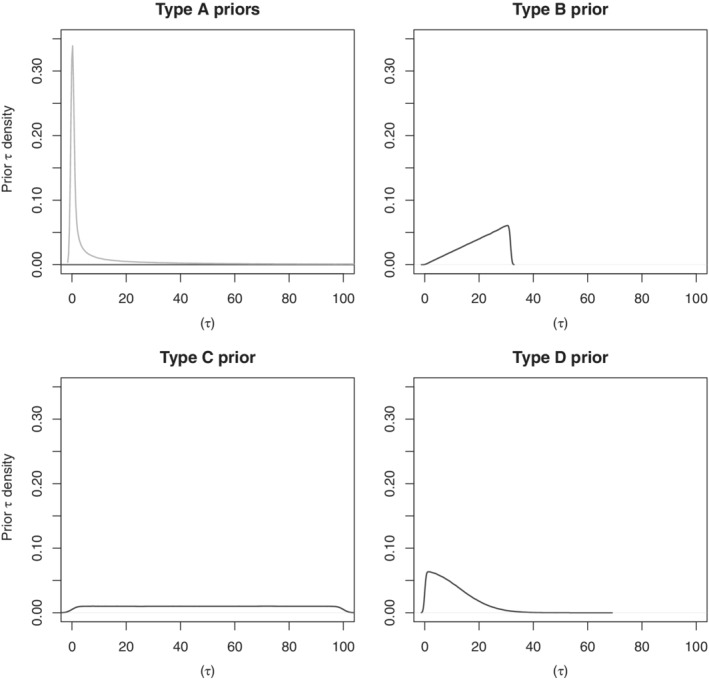
Prior distributions classified by their density shapes. Type A priors include both *Gamma*s on *v*_*τ*_ (AG, ag) and the less restrictive *Uniform*(−10, 10) on log(*τ* ^2^) (AU) The *Gamma* prior has a very small peak near zero, while the peak of the *Uniform* type A prior is higher both support very large *τ*‐values. Type B priors include both *Uniform* on *τ* ^2^ (B, b), Type C priors include the *Uniform*s on *τ* priors (C, c), Type D include both the *Half*‐*normal* on *τ* priors (DN, dn) and the more informative *Uniform*(−10, 1.386) on log(*τ* ^2^) prior (du). The less restrictive options per considered prior are presented in this figure, while the more informative options within each prior retain a similar shape but cover a smaller range of values, except for the *Uniform*(−10, 1.386) on log(*τ* ^2^) prior. This prior results in a form closer to Type D priors. For clarity of results the *x* − *axis* is graphically truncated for values larger than 100. Figure 3 in Data S1 provides a comparison between the less and more restrictive prior options

While conducting a meta‐analysis, the estimation of heterogeneity is rarely of primary interest. In cases of small and sparse meta‐analyzes, estimation of *τ* can quickly become infeasible. Therefore, the choice of heterogeneity priors shall also be driven by its ability to aid the proper estimation of the treatment effect. Different priors have been suggested in the literature, for several functions of *τ* (Table 2). In such sparse settings, the impact and behavior of each prior is based primarily on its distributional shape. Therefore, a sensible manner of clustering such priors would be to evaluate the way they distribute prior mass on the same scale, that is, on *τ* scale. In this context, priors can be clustered in, at least, the following four groups. First, Type A priors place more mass close to 0 but support very large values of *τ* as well^13^, ^15^ (see Figure 1). This type of priors contain the *Gamma*(*α*, *β*) prior distributions (AG, ag) on the precision (*v*_*τ*_ = 1/*τ* ^2^) and the less restrictive prior on *Uniform*(−10, 10) on the log(*τ* ^2^) scale (AU). Type B priors place more mass in larger values of *τ*; that is, *Uniform* on *τ* ^2^ scale (C, c). Type C priors place mass uniformly in a selected range of *τ* (ie, *Uniform* on *τ* scale [B, b]). Finally, Type D priors place most of the mass in small values of *τ* but they naturally bound the prior range to more plausible values than Type A priors. Examples of Type D priors are the *Half*‐*normal* priors (DN, dn) on *τ* and the more informative prior version of *Uniform*(−10, 1.386) on the log(*τ* ^2^) (du). Type D prior distributions are advocated for MA of a few trials.^13^, ^19^, ^27^, ^28^ Within each prior we examine two options based on the informativeness provided by their hyper‐parameters, one less restrictive (AG, AU, B, C, DN) and one more restrictive (ag, b, c, dn, du) alternative (Table 2).

**TABLE 2:** Description of considered heterogeneity (*τ*) priors for a Bayesian meta‐analysis

ID ‐ Abbr.	*g*(*τ*)	∼*f*(.)	Restrictive	*τ* Median	*τ* (95% range)
AG	1/*τ* ^2^	∼*Gamma*(0.001, 0.001)	Less	>100	(>100, +∞)
ag	1/*τ* ^2^	∼*Gamma*(0.1, 0.1)	More	0.3	(12.9, > 100)
AU	log(*τ* ^2^)	∼*Uniform*(−10, 10)	Less	1	(0.01, > 100)
du	log(*τ* ^2^)	∼*Uniform*(−10, 1.386)	More	0.1	(0.01, 1.7)
B	*τ* ^2^	∼*Uniform*(0, 1000)	Less	22.4	(5, 31.2)
b	*τ* ^2^	∼*Uniform*(0, 4)	More	1.4	(0.3, 2)
C	*τ*	∼*Uniform*(0, 100)	Less	50	(2.5, 97.5)
c	*τ*	∼*Uniform*(0, 2)	More	1	(0.05, 1.95)
DN	*τ*	∼*Half*‐*normal*(0, 100),	Less	6.75	(0.3, 22.4)
dn	*τ*	∼*Half*‐*normal*(0, 1),	More	0.7	(0.03, 2.24)
E	*s*_0_/(*s*_0_ + *τ*)	∼*Uniform*(0, 1)	—	—	—
e	*τ* ^2^	∼*Half*‐*normal*(0, Φ[0.75]/*s*_0_),	—	—	—

*Note:* $s_{0}=\sqrt{k/\sum \left(s_{i}^{-2}\right)}\;$ and $\;s_{i}^{2}$ are the within‐study variances. ID ‐ Abbr.: Identification letter and abbreviation for each prior.

Finally, we use the estimates of the within‐study variances ($s_{i}^{2}$) to examine two data‐driven priors (E, e) that both incorporate the harmonic mean ($s_{0}=\sqrt{k/\sum \left(1/s_{i}^{2}\right)}\;,i=1,2,\ldots ,k$) of the $s_{i}^{2}$ of the trials included in the MA.^20^, ^29^ More specifically, prior E, also known as the *DuMouchel* prior has been suggested for very small sample sizes and, by utilizing *s*_0_, it induces shrinkage on the *τ* prior distribution.^30^ Small values of *s*_0_ result in a narrow‐tailed prior distribution on *τ* and more shrinkage, while large values of *s*_0_ result in a wide‐tailed prior distribution on *τ* and less shrinkage.

In the following section we introduce two motivating examples, illustrate the results when different priors are used and discuss the implications.

## 3 MOTIVATING EXAMPLES

Multifocal motor neuropathy is a progressive rare disorder in which the muscles weaken gradually. Multifocal motor neuropathy is not often fatal but can lead to a significant degree of disability for the patient. Prevalence is estimated at 1‐2 cases per 100 000.^31^ A literature review and MA assessed the efficacy and safety of intravenous immunoglobulin in multifocal motor neuropathy.^3^ The same evidence was presented in the European Medicines Agency Public Assessment Report of Kiovig.^32^ The primary outcome was the improvement in disability scale using MRC (Medical Research Council) scores that evaluate the muscle strength. Three two‐arm studies reported the outcome, accounting for a total of 36 recruited patients with seven reported events in the intravenous immunoglobulin arm and two in the placebo arm. The original MA reported no heterogeneity.^3^

For the second example, we consider Guillain‐Barre syndrome with a MA of four available studies. Guilen‐Barre syndrome has a prevalence of 1‐9 cases per 100 000^31^ and refer to a number of rare post‐infection neuropathies. A literature review and MA summarized RCTs that compared intravenous immunoglobulin to control (plasma exchange).^4^ For one of the secondary outcomes, treatment discontinuation, a few arms reported zero events. This example has been used for evaluating a number of heterogeneity estimators under the inverse‐variance method and has been shown to produce conflicting inferences.^21^ Data for both examples are illustrated in Table 3.

**TABLE 3:** Motivating examples; (A) Efficacy endpoint: Improvement in disability, Therapy: Intravenous immunoglobulin vs Placebo, Condition: Multifocal motor neuropathy (B) Efficacy endpoint: Treatment discontinuation, Therapy: Intravenous immunoglobulin vs Plasma Exchange, Condition: Guillain‐Barre syndrome

(A) Multifocal motor neuropathy ‐ Improvement in disability^3^							(B) Guillain‐Barre syndrome ‐ Treatment discontinuation^4^						
Author	r_iT_	*n*_*iT*_ − *r*_*iT*_	r_iC_	*n*_*iC*_ − *r*_*iC*_	${\hat{\pi }}_{i.}$	*w*_*i*, *in*_	Author	r_iT_	*n*_*iT*_ − *r*_*iT*_	r_iC_	*n*_*iC*_ − *r*_*iC*_	${\hat{\pi }}_{i.}$	*w*_*i*, *in*_
Azulay	0	5	0	5	0	–	Meche	0	74	12	61	0.08	0.39
Berg	3	3	0	6	0.25	0.20	Bril	0	26	0	24	0	–
Lger	4	3	2	5	0.43	0.80	PSGBS	3	127	18	103	0.09	0.58
							Nomura	1	22	1	23	0.04	0.03

*Note: r*_*i*, *j*_ event in control/treatment group, *n*_*i*, *j*_ − *r*_*i*, *j*_ non‐event in control/treatment group ${\hat{\pi }}_{i.}$ = observed probability of event in each trial, *w*_*i*, *in*_ = weight of initial analysis.

### 3.1 Analysis of motivating examples

**FIGURE 2:**
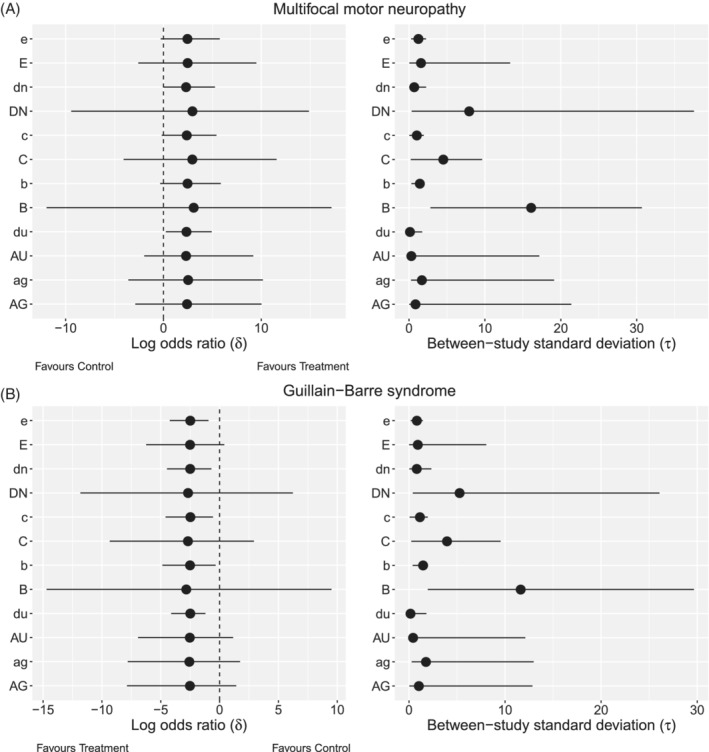
Posterior medians and 95% credible intervals of the overall effect (log odds ratio) and the between‐study SD (*τ*) for the two motivating examples (A) Multifocal motor neuropathy and (B) Guillain‐Barre syndrome. (AG, ag) ‐ *Gamma* on *v*_*τ*_, (AU, du) ‐ *Uniform* on log(*τ* ^2^), (B, b) ‐ *Uniform* on *τ* ^2^, (C, c) ‐ *Uniform* on *τ*, (DN, dn) ‐ *Half*‐*normal* on *τ*, (e) *Half*‐*normal* on *τ* ^2^, (E) ‐ *DuMouchel* prior. (AG, AU, B, C, DN) are less restrictive priors on *τ* and (ag, dn, b, c, dn) are more informative priors on *τ*

A robust choice of prior is not trivial for our examples. To examine the behavior of the priors, we use Rjags^33^, ^34^ to fit three chains of 850 000 samples after a burn‐in of 150 000 samples and a thinning interval of 35 samples for each model. Figure 2 presents the posterior median (as a point estimate) and credible intervals of *δ* and *τ* for the two motivating examples under different priors. The letters in Figure 2 correspond to the letters in Table 2.

The choice of prior for *τ* has substantial impact on the posterior credible intervals for *δ*. The posterior median for *δ* varies substantially as well. More specifically, in the multifocal motor neuropathy example, the posterior median *δ* has a range of (2.31, 3.27) depending on the *τ* prior choice (Figure 2A). In the Guillain‐Barre syndrome example, the posterior median *δ* has a range of (−2.52, −2.80) (Figure 2B). The posterior mean of *δ* in both examples shows even greater diversity. Interval estimation of *δ* also varies substantially. Different priors and types of priors lead to considerably divergent inference (Figure 2). All Type A priors show a similar behavior upon the estimation of *δ* in both examples.

## 4 SIMULATION STUDY

To incorporate heterogeneity successfully in both study arms, we simulated study‐specific logits for each arm, following the simulation strategy of Hartung and Knapp (Reference 35, *pRandom* in Reference 36). Hence, we assumed an initial fixed event probability in the control group and we calculated the event probability in the treatment group, based on a true overall treatment effect. Further, we simulated study‐specific logits from a normal distribution with between‐study SD equal to $\tau /\sqrt{2}$ for the control and treatment arm. We utilized the simulated logits to compute the study‐arm event probabilities by back‐calculating and finally we simulated events for each study arm.^36^

We evaluated a number of scenarios by varying the number of trials (*k*), the number of patients per trial arm (*n*_*ij*_), the control event rate (*π*_*c*_), the between‐study heterogeneity (*τ*) and the overall treatment effect (*δ*). More specifically, the number of trials varied as *k* ∈{2, 4, 6} while we assumed equal number of patients per trial arm (*n*_*iC*_ = *n*_*iT*_) and uniformly sampled either between 40 to 50 or between 5 to 10. These sample sizes were selected to represent realistic scenarios for efficacy and safety endpoints of rare and ultra‐rare diseases.^2^ The control event rate (*π*_*c*_) in each trial took set values as follows; very low event rate (0.05), low event rate (0.1), moderate event rate (0.3). Specific combinations of sample size and control group event rates lead to particular percentages of zero‐event trials in MAs of the simulated data (Data S1 ‐ Table 1). The between‐study SD took values between *τ* ∈{0.01, 0.5, 1}. Finally, we examined three values for the overall treatment effect on the logOR scale, *δ* ∈{0, 0.5, 3}.

First, the 12 clustered priors above are evaluated for all scenarios and then a number is selected for further evaluation. Therefore, the number of scenarios is in total 1994. For each scenario we generated 1000 simulated datasets. We performed simulations using JAGS^33^ and R^37^ via a High Performance Cluster. We fitted every model via three parallel chains of 30 000 samples, a burn‐in of 4500 samples and a thinning interval of three samples.

In sparse settings the parameters' Markov chain Monte Carlo sampling convergence is of concern. We conducted selective convergence checks on the Markov chain Monte Carlo algorithms via trace plots, convergence diagnostics via the CODA package^38^ and focused on the most extreme scenarios of sparsity. We fitted every model via three parallel chains and we accounted for autocorrelation by applying a thinning interval of five samples. Overall, convergence was achieved. We analytically report on diagnostics in the Data S3, where we compare the convergence of different priors. Diagnostic assessment was performed for both the examples (via generation of 1,000,000 Markov chain Monte Carlo samples) and the simulation study (via generation of 34,500 Markov chain Monte Carlo samples).

Each scenario was mainly evaluated by the following performance measures: (a) average posterior median for *δ*, (b) coverage of the 95% credible interval (CrI). We also report and discuss the mean square error of *δ* and the average posterior median estimates of *τ* for exploratory purposes and completeness. Prior robustness was defined by adequate overall measures and small observed fluctuations in coverage of the 95% credible interval among the scenarios considered.

## 5 RESULTS OF SIMULATION STUDY

**FIGURE 3:**
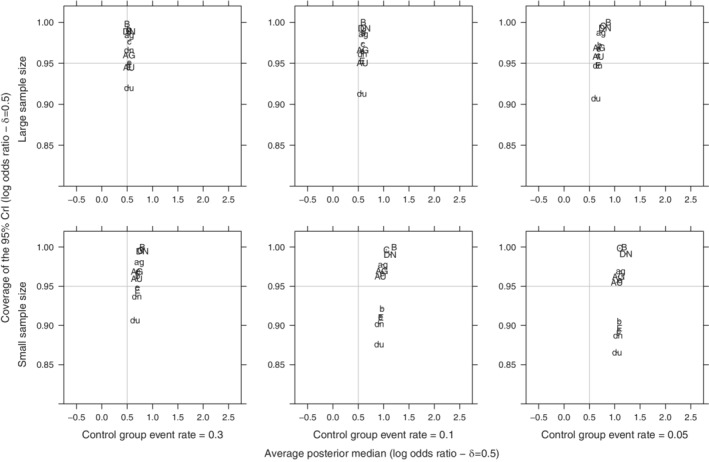
Scatter plot of average posterior median overall effect (log odds ratio) against its mean coverage of the 95% CrI for all simulated scenarios (Overall effect: *δ* = 0.5, between‐study SD: *τ* ∈{0.01, 0.5, 1}, number of trials: *k* ∈{2, 4, 6}) of a meta‐analysis with control group event rate: *π*_*c*_ ∈{0.05, 0.1, 0.3} with small sample size trials (*n*_*ij*_ ∼ *Uniform*[5, 10]) or large sample sized trials (*n*_*ij*_ ∼ *Uniform*[40, 50]). (AG, ag) ‐ *Gamma* on *v*_*τ*_, (AU, du) ‐ *Uniform* on log(*τ* ^2^), (B, b) ‐ *Uniform* on *τ* ^2^, (C, c) ‐ *Uniform* on *τ*, (DN, dn) ‐ *Half*‐*normal* on *τ*, (e) *Half*‐*normal* on *τ* ^2^, (E) ‐ *DuMouchel* prior. (AG, AU, B, C, DN) are less restrictive priors on *τ* and (ag, dn, b, c, dn) are more informative priors on *τ*

**FIGURE 4:**
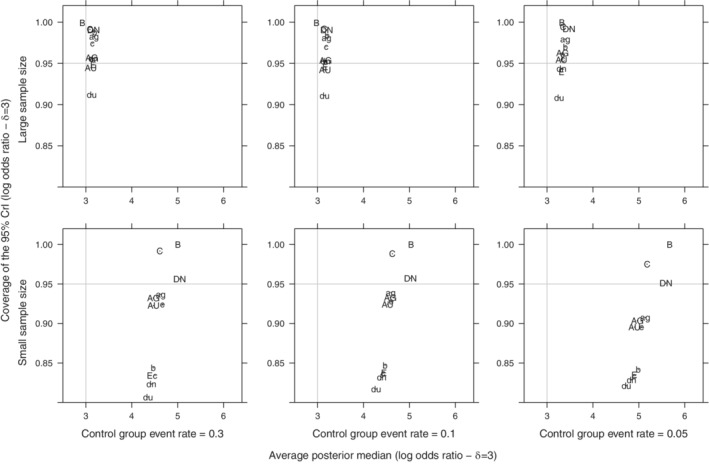
Scatter plot of average posterior median overall effect (log odds ratio) against its mean coverage of the 95% CrI for all simulated scenarios (Overall effect: *δ* = 3, between‐study SD: *τ* ∈{0.01, 0.5, 1}, number of trials: *k* ∈{2, 4, 6}) of a meta‐analysis with control group event rate: *π*_*c*_ ∈{0.05, 0.1, 0.3} with small sample size trials (*n*_*ij*_ ∼ *Uniform*[5, 10]) or large sample sized trials (*n*_*ij*_ ∼ *Uniform*[40, 50]). (AU, Au) ‐ *Gamma* on *v*_*τ*_, (AU, du) ‐ *Uniform* on log(*τ* ^2^), (B, b) ‐ *Uniform* on *τ* ^2^, (C, c) ‐ *Uniform* on *τ*, (DN, dn) ‐ *Half*‐*normal* on *τ*, (e) *Half*‐*normal* on *τ* ^2^, (E) ‐ *DuMouchel* prior. (AG, AU, B, C, DN) are less restrictive priors on *τ* and (ag, dn, b, c, dn) are more informative priors on *τ*

For relatively large sample sizes and higher *π*_*c*_, regarding the posterior estimation of *δ*, all priors perform similarly (Figures 3 and 4).The overall performance of the priors deteriorates at a low control group event rate (*π*_*c*_ = 0.05) for a few small RCTs MA, as the average posterior median of *δ* is overestimated (Figures 3 and 4) at all levels of true heterogeneity. Furthermore, we observe an overall positive bias in the posterior median estimation of *δ*, when *δ* is large.

All Type A priors retain more robust 95% coverage in comparison to other prior groups (Figures 3 and 4). More specifically, the *Uniform*(−10, 10) on log(*τ* ^2^) scale prior (AU) retains a more robust 95% coverage at small values of *π*_*c*_, independently of sample size and it properly estimates the posterior median logOR on average as well (Figures 3 and 4). The *DuMouchel* empirical prior (E) shows a comparable behavior. The 95% coverage of Type B, C and D priors varies throughout the evaluated scenarios from conservative in larger sample sizes to liberal 95% coverage in smaller sample sizes (Figures 3 and 4). All priors encounter issues regarding the 95% coverage when the treatment effect is large (*δ* = 3), the sample size is limited and the control event rate is very small (*π*_*C*_ = 0.05) (Figure 4).

More informative priors for *τ* (b, c, dn, du) tend to produce a less variant posterior point estimate of *δ*, while less restrictive priors that mostly support larger values for *τ* (B, C, DN) tend to overestimate *δ* heavily (Figures 3 and 4). Moreover, the use of the latter group of priors at any level of *π*_*c*_ results in conservative inference for *δ* (Figures 3 and 4). This set of less restrictive priors and (du) prior, a prior that also has the smallest prior *τ* median (Table 2), all four priors performed poorly in terms of 95% coverage irrespective of the sparseness of events.

It should be noted that even though Figures 3 and 4 only provide a general view of all simulated scenarios, we did not observe deviations regarding the average posterior median of the overall treatment effect (*δ*) when investigating specific scenarios. Additional averaged and scenario‐specific simulations are presented in Data S2.

**FIGURE 5:**
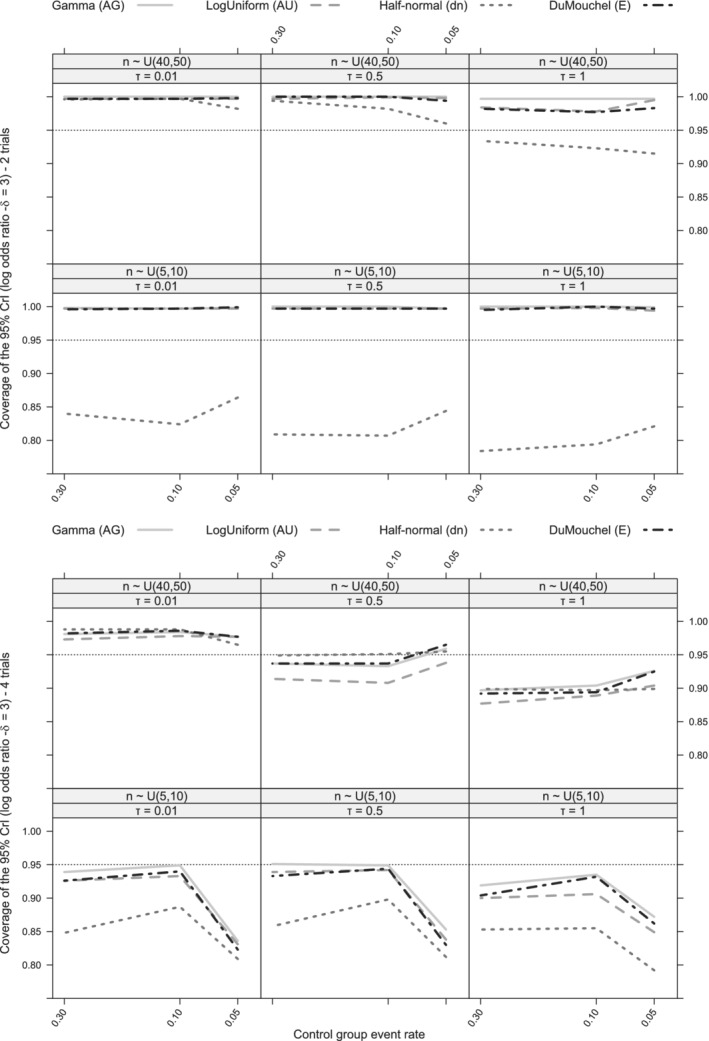
Coverage of the 95% CrI line plots of the overall effect (log odds ratio) on different control group event rate levels for a large true overall effect (*δ* = 3), three values of *τ* ∈{0.01, 0.5, 1} and small sample size trials (*n*_*ij*_ ∼ *Uniform*[5, 10]) or large sample sized trials (*n*_*ij*_ ∼ *Uniform*[40, 50]). (AG): *Gamma*(0.001, 0.001) on *v*_*τ*_, (AU): *Uniform*(−10, 10) on log(*τ* ^2^), (dn): *Half*‐*normal*(0, 1) on *τ*, (E): *DuMouchel* prior. Results for 6 trials can be found in Data S2

**FIGURE 6:**
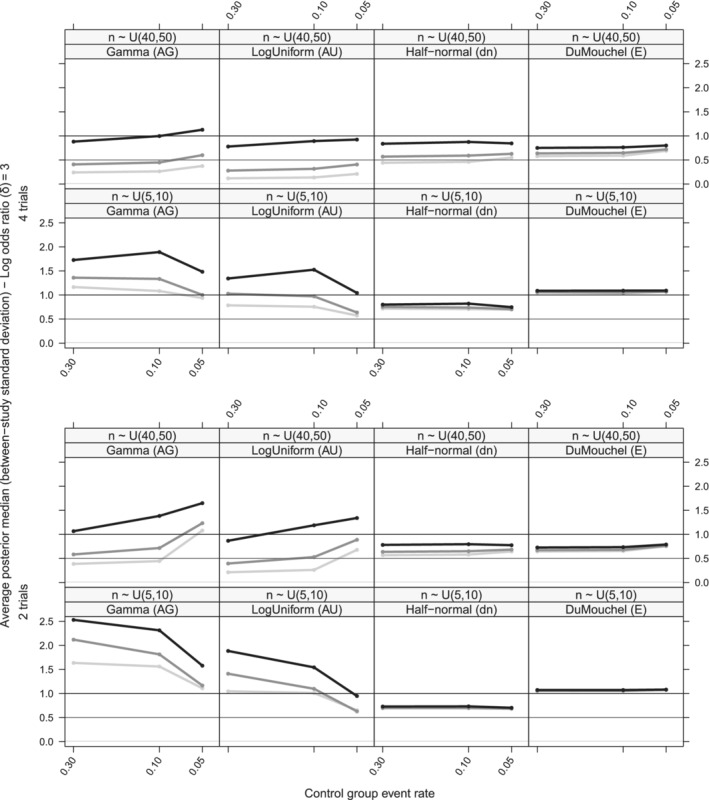
Average posterior median line plots of the between‐study SD (*τ*) on different control group event rate levels for a large true overall effect (*δ* = 3) and small sample size trials (*n*_*ij*_ ∼ *Uniform*[5, 10]) or large sample sized trials (*n*_*ij*_ ∼ *Uniform*[40, 50]). The gray lines represent 3 levels of heterogeneity, namely, light gray: *τ* = 0.01, gray: *τ* = 0.5, dark gray: *τ* = 1 (AG): *Gamma*(0.001, 0.001) on *v*_*τ*_, (AU): *Uniform*(−10, 10) on log(*τ* ^2^), (dn): *Half*‐*normal*(0, 1) on *τ*, (E): *DuMouchel* prior. Results for 6 trials can be found in Data S2

For clarity of results, after studying all priors (Figures 3, 4 and Data S2), we focus on four priors (AG, AU, dn, E) which either (1) performed more robustly in the current simulation study (AU, E), (2) are commonly used in the literature (AG) and/or (3) have been suggested in recent literature for meta‐analysis of rare diseases (dn).^9^ We present selected scenarios for *δ* = 3 in the main manuscript (Figures 5 and 6).

### 5.1 Coverage of the 95% CrI for the overall treatment effect (*δ*)

The value of the treatment effect does not heavily affect the coverage of the 95% CrI. Specifically, for a MA of four trials, most robust coverage is generally produced by the two Type A priors, the *Gamma*(0.001, 0.001) prior on *v*_*τ*_ (AG) and (AU), alongside with (E) empirical prior (Figure 5). However, in a MA of less than four trials, prior (AG) induces systematically larger deviations from the nominal 95% coverage in comparison to priors (AU) and (E) (Figure 5). The Type D *Half*‐*normal*(0, 1) prior (dn) prior either induce (1) over‐coverage for low levels of true heterogeneity (*τ* ≤ 1) or low event rates or (2) large under‐coverage for large true heterogeneity (*τ* = 1), regardless of the event rate. In comparison to the three priors described above, the (dn) prior shows the least robust coverage throughout all scenarios and more particularly for varying sample sizes or levels of *τ* (Figure 5 and Data S2).

### 5.2 Mean square error of the overall treatment effect (*δ*)

All priors produce comparable levels of mean square error (Data S2 ‐ Figures 1‐3 and 12‐15). The priors that produce the least optimal and most divergent behavior in comparison to the rest are the (DN) and (B) priors.

### 5.3 Exploring the heterogeneity estimate behavior (*τ*)

All 12 priors produced biased results. In less sparse scenarios (*n*_*ij*_ ∼ *U*[40, 50]), *k* = 4, 6) the type A priors (AU) and (AG) show the least bias on *τ*, irrespective of the true heterogeneity level (Figure 6 and Data S2). Prior (dn), behaved similarly to all other more informative prior choices and showed difficulty in identifying any level of true heterogeneity (Figure 6).

## 6 REVISITING THE MOTIVATING EXAMPLES

**FIGURE 7:**
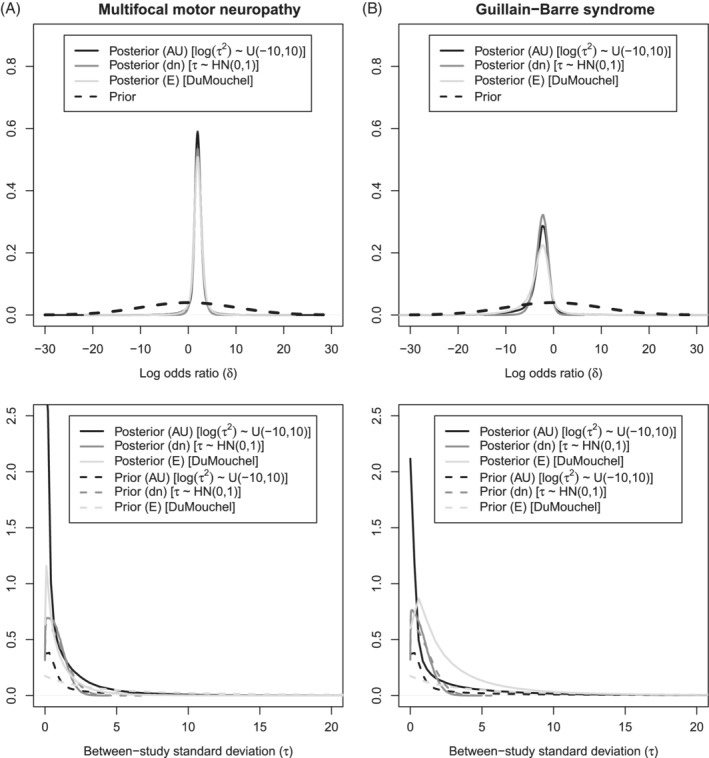
Posterior summaries for the overall effect (*δ*) and the between‐study SD (*τ*) of the Multifocal motor neuropathy and Guillain‐Barre syndrome examples for (AU): *Uniform*(−10, 10) on log(*τ* ^2^), (dn): *Half*‐*normal*(0, 1) on *τ* and (E): *DuMouchel* empirical prior. The analyzes are based on 850 000 iterations with a burn‐in of 150 000 iterations and a thinning interval of 35 iterations

Following the results of the simulation study, prior type A *Uniform*(−10, 10) on the log(*τ* ^2^) prior (AU) is preferred for the Guillain‐Barre syndrome example (four trials, low event rates, relatively large sample size). When we apply this prior, the primary inference of these studies would produce a posterior probability of *δ* > 0 equal to 96%. This is less than the 99% posterior probability which is produced by the Type D *Half*‐*normal*(0, 1) prior (dn) on *τ*, a prior that showed non robust overall but sufficient coverage at low to moderate *π*_*c*_ combined with low to moderate *τ* settings (Figure 5). Therefore, inference with both priors suggests efficacy of intravenous immunoglobulin in comparison with plasma exchange in terms of treatment discontinuation and result in comparable posterior distributions (Figure 7) and medians for the logOR (*δ*_*AU*_ = −2.51 ‐ *δ*_*dn*_ = −2.49).

Likewise, for the more sparse multifocal motor neuropathy example (3 trials, moderate event rates, relatively small sample size), prior (AU) would also be preferred. When we apply this prior, the primary inference for these studies would produce a posterior probability of *δ* > 0 equal to 93%, but when prior (dn) is applied, the posterior probability becomes 97%, which would have overstated our confidence in the effectiveness of intravenous immunoglobulin regarding improvement in MRC scale, based on results of the simulation study. Similarly to the Guilen‐Barre syndrome case study, relying on priors (AU) or (dn) produces comparable posterior median logORs (*δ*_*AU*_ = 2.32 ‐ *δ*_*dn*_ = 2.31), as expected by the reported simulation study (Figures 3 and 4).

In both examples, data‐driven prior (E) produces similar probability statements and posterior median logORs to the Type A (AU) prior, a behavior which is aligned with the results of the simulation (Figure 5). Based on the simulation study, a Type A prior (ie, AU) that showed robust 95% coverage should be chosen as it provides less variable behavior in comparison to the studied alternatives under both known and unknown parameters (Types B, C and D), as well.

A comparison between the two priors that performed robustly through the simulation study (AU and E) and the commonly used half‐normal prior (dn) is presented in Figure 7 for the multifocal motor neuropathy and Guillain‐Barre syndrome examples respectively. In both examples, when prior (AU) is applied, the posterior distribution of *τ* differs considerably from its prior. However, when prior (dn) is applied, the posterior distribution of *τ* becomes more prior‐driven, a behavior which is also depicted in our simulation (Figure 6). In Data S1 (Table 2), interested readers can find the extended results of all considered prior choices.

## 7 MAIN FINDINGS

- The choice of type of prior and prior distribution for *τ* heavily influences not only the posterior mean/median estimates of *τ* but also the posterior mean/median estimates of *δ* in a sparse‐events MA of a few small trials.
- In a sparse meta‐analysis of a few small (*n*_*ij*_ ∼ [5, 10]) studies, priors that place most of the mass in small values of *τ* but naturally restrict the range to more plausible values (D) (ie, dn, du) should be avoided as they do not provide robust point and proper interval estimation of *δ*.
- Type A priors that place more mass on small values without excluding very large *τ* prior values (e.g. AU) are suggested as a robust choice for a sparse‐events MA of a few small trials.
- In many scenarios and even for very sparse settings, the Type A prior *Uniform*(−10, 10) on the log(*τ* ^2^) scale prior (AU) shows good coverage overall combined with less overestimation of *δ* or *τ* in comparison to other prior choices. The *DuMouchel* prior (E) shows a similar behavior.
- The less restrictive choices of priors that place mass uniformly in a selected range (B) and/or priors that place more mass in larger values of *τ* (C) and the empirical prior (DN) are not appropriate for a sparse‐events MA of a few small trials, as they overestimate *τ* and produce conservative inferences, while resulting in improper estimation of *δ*. Their more informative alternatives (b, c and dn) produce more reliable inferences at high *π*_*c*_, but they result in liberal inferences when combined with large true heterogeneity (*τ* = 1) and low *π*_*c*_. All six prior choices have difficulties to identify varying levels of *τ*.

## 8 DISCUSSION

Based on previous research, it is generally accepted that the choice of prior distribution on *τ* largely impacts the posterior interval estimation of *δ* in a meta‐analysis of a few small trials.^9^, ^19^, ^20^, ^28^ We demonstrated that in very sparse settings measures, such as the overall posterior median of *δ*, can become very inconsistent under alternative priors on *τ* as well. Even though, the final choice of prior should take into account the specific characteristics of each conducted meta‐analysis, a solution in such sparse conditions would be to identify prior shapes that show robustness in the operational features of the posterior estimation of *δ*.

In this study we demonstrated that priors which place mass on small values of *τ* but sufficiently support larger values as well (Type A priors, eg. AU ‐ *Uniform*(−10, 10) prior on log(*τ* ^2^) scale) showed on average robust behavior in most scenarios, followed by *DuMouchel* empirical prior (E), in comparison to other choices. Type D priors such as the dn ‐ *Half*‐*normal*(0, 1) on *τ*, a prior that has been compared under an approximate normal setting and has been evaluated in settings of a few small trials,^9^, ^10^ did not perform satisfactorily neither under large levels of true heterogeneity nor under different settings of trial size and number of trials. Type A priors and *DuMouchel* empirical prior place larger uncertainty around *τ* (Table 2) and produce a more data‐driven inference on *δ*, in comparison to Type D priors such as the *Half*‐*Normal* (dn) prior or the more informative *Uniform*(−10, 1.386) on log(*τ* ^2^) scale prior (du), which produces a more prior‐driven inference on *δ*. Furthermore, we demonstrated that the use of priors with either less restrictive or very confining prior range may be equally problematic, in terms of operational features and robustness.

### 8.1 Findings in perspective

Our study extends previous research on Bayesian hierarchical models' evaluations^19^, ^20^, ^28^ in sparse‐events MA of small populations. Contrary to previous evaluations on priors for heterogeneity,^9^, ^10^, ^19^, ^28^ we focused on a sparse‐event setting, we then grouped the evaluated priors based on their shape. Except for observing the expected variations in the posterior intervals of *δ*, we observed a variation in the posterior medians of *δ* as well. Namely, priors that favor small *τ* are the ones that misestimate *δ* the least at very low event rates.

We further noticed a general overestimation when *δ* is large, as well as to a smaller extent when *δ* takes smaller values. The primary reason for the overestimation of *δ* is the nature of a dichotomous outcome. For positive *δ*, more events are observed in the treatment arm, especially when *δ* is large.^36^ Events in the treatment arm combined with zero events in the control arm result in overestimation. We also applied an alternative model that applies larger variance to log*it*(*π*_*T*_) than to log*it*(*π*_*C*_) in comparison to model (1) and Model 2 in Reference 24. Conclusions remained comparable, though when the alternative model was applied an underestimation of *δ* was observed when *π*_*c*_ was very low.

The variance within a single study relative to the estimated heterogeneity between studies determines this study's impact on the overall inference for *δ*. Naturally, small studies with zero events would produce a large within‐study variability (standard errors) around the logOR study‐specific effect which decreases the study's impact on the posterior overall effect. However, prior distributions that favor large values for *τ* allow small studies to have a larger weight. As a result, the contribution of small studies with one or two reported zero arms in a MA is enhanced when considering priors that support large *τ*. In both examples we reviewed herein, the increasing weight of studies with no observed events, mostly in a single arm, explains why the posterior median of *δ* are overestimated when less restrictive priors are applied (Figure 2 and Data S1 ‐ Table 2). Therefore, in combination with the observed unstable study‐specific treatment effect issues, alternative prior assumptions may enhance the impact of zero events in a few small trials MA, inducing a “small MA zero‐event” bias on *δ*.

### 8.2 Main limitations

This work is subject to the assumption of normality for the study‐specific effects and the overall treatment effect, by placing a weakly diffused normal prior on *δ*_*i*_ and *δ*; instead other dependence structures between *δ* and *δ*_*i*_ may be preferred.^39^ Despite its common use, this assumption may not be appropriate considering the small number of studies and sparsity of events. Model 1 further assumes that the log*it*(*π*_*iT*_) and log*it*(*π*_*iC*_) have equal variances. This can be a restrictive assumption for which alternatives have been discussed.^40^ In addition, other priors on *μ*_*i*_, *δ*_*i*_ or *δ* may be considered; namely, a *Uniform*, a *Student*‐*t*, a *Truncated*‐*t* or a *Cauchy* prior.^41^ After partially evaluating these options through simulation, we did not observe changes in our conclusions. In the setting of a few small trials, informative empirical priors that are based on published MAs of the Cochrane database can be used in a new MA of binary outcomes.^42^, ^43^ However, such empirical priors have not been yet tailored for meta‐analyzes in rare diseases and therefore, may not be representative of heterogeneity commonly observed in such cases.^43^, ^44^, ^45^ Based on preliminary non‐reported results, such priors are expected to result in suboptimal frequentist characteristics similar to the very informative priors studied herein. Another restrictive option, given the small sample sizes, would be to model the studies as covariates and avoid the normal random‐effects assumption.

In the simulation study we focused on positive treatment effects with low control event rates but not negative treatment effects with larger control event rates assuming that such effects are symmetric and their probabilities of success are reversed between the treatment arms.

The behavior of a Bayesian MA might depend on the type of binary effect measure (log odds ratio, log risk ratio, risk difference). Such alternative measures could be of importance with sparse events MAs when normal approximations do not hold or when the logOR is undefined.^6^, ^7^

Finally, one should consider the issue of inefficient Markov chain Monte Carlo sampling for rare events.^46^, ^47^ In such extremely sparse settings, our findings might be sensitive to the sampling engine of the simulation study. Regardless of the sampler applied, we recommend conducting a formal convergence analysis in such sparse settings.

## 9 CONCLUSION

To conclude, a random‐effects MA using a Bayesian binomial‐normal hierarchical model has the potential to deal with large numbers of zero events. The sensitivity of Bayesian models to the choice of priors is confirmed and produces not only diverse credible intervals but also diverse posterior medians for the overall treatment effect (*δ*). We showed that when performing a Bayesian binomial‐normal MA under such sparse conditions, robust priors should have more mass close to zero, while supporting very large values as well (ie, a less informative *Uniform*(−10, 10) prior on log(*τ* ^2^)). Priors that support only large or only mainly small values of heterogeneity (*τ*) result in substantial misestimation of *δ* in such sparse settings and should be avoided. Aside from robustness researchers should aim to account for the specific characteristics of each conducted meta‐analysis before choosing a prior and setting prior levels of expected heterogeneity.

## CONFLICT OF INTEREST

The authors declare no potential conflict of interest.

## AUTHOR CONTRIBUTIONS

Konstantinos Pateras, Stavros Nikolakopoulos and Kit C. B. Roes conceived the idea and designed the study, Konstantinos Pateras undertook all the simulation analyzes. Konstantinos Pateras drafted the paper and further revised given critical comments from Stavros Nikolakopoulos and Kit C. B. Roes. All authors have read and accepted the final manuscript.

## Supporting information


**Data S1.** General tables and figures.

**Data S2.** Simulation figures.

**Data S3.** Diagnostics.

## Data Availability

The data used in the examples of the article can be found directly from articles included in the references (3; 4). The simulated datasets and corresponding R/JAGS code that supports the findings of this study are available online (48).
