# Supplementary material for: Prior distributions for variance parameters in a sparse‐event meta‐analysis of a few small trials
Source: Pharm Stat. 2020 Aug 6;20(1):39–54. doi: 10.1002/pst.2053 (PMC7818503; doi:10.1002/pst.2053)
Supplement: Supplementary file 1 — Data S1. General tables and figures. [file PST-20-39-s001.pdf]

Supplementary material SI - General tables and figures

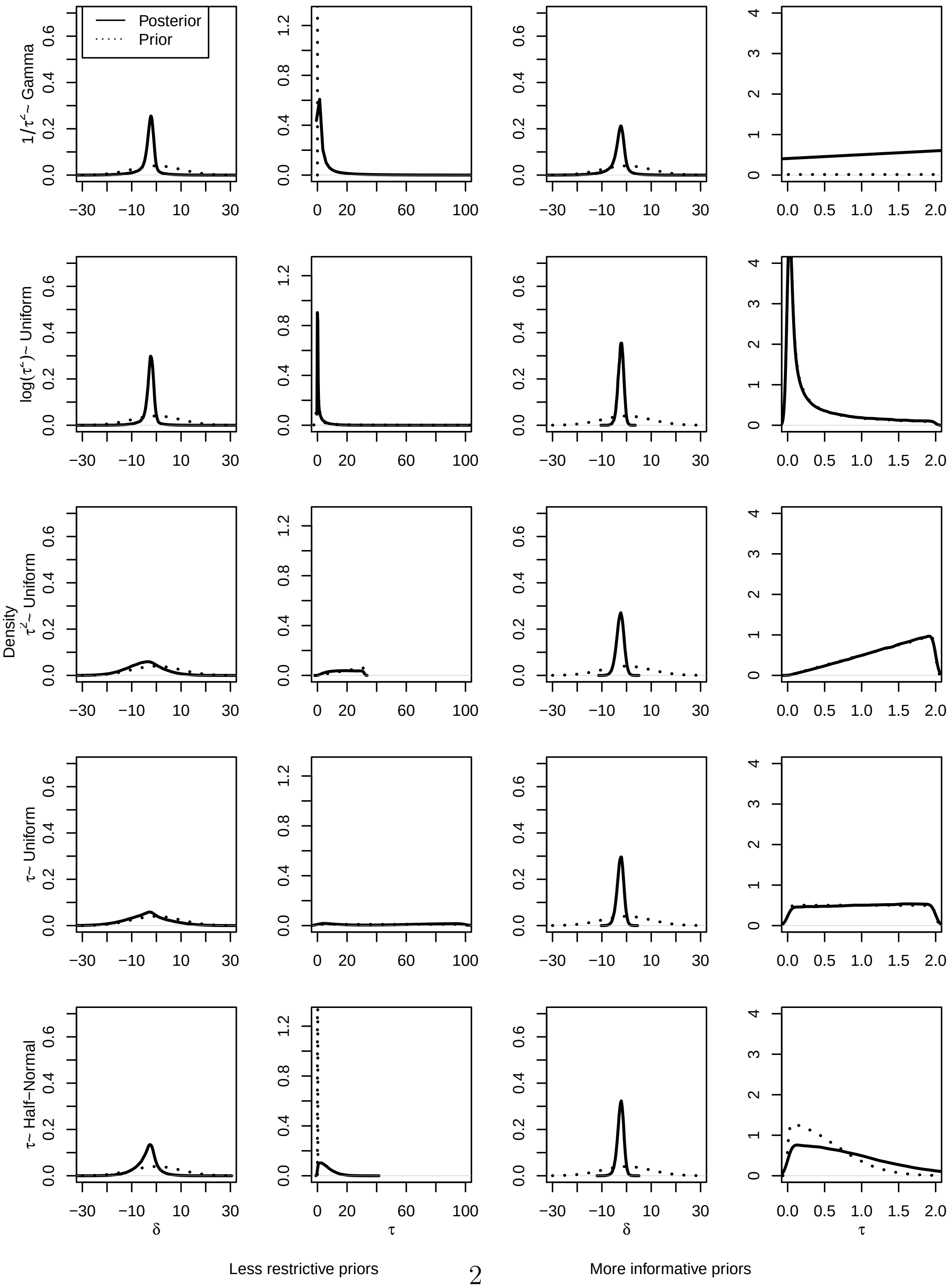

Figure 1: Priors and posterior distributions of the 10 non-empirical heterogeneity priors for the overall treatment effect (δ) and the between-study standard deviation (τ) applied on the Multifocal motor neuropathy example 1.

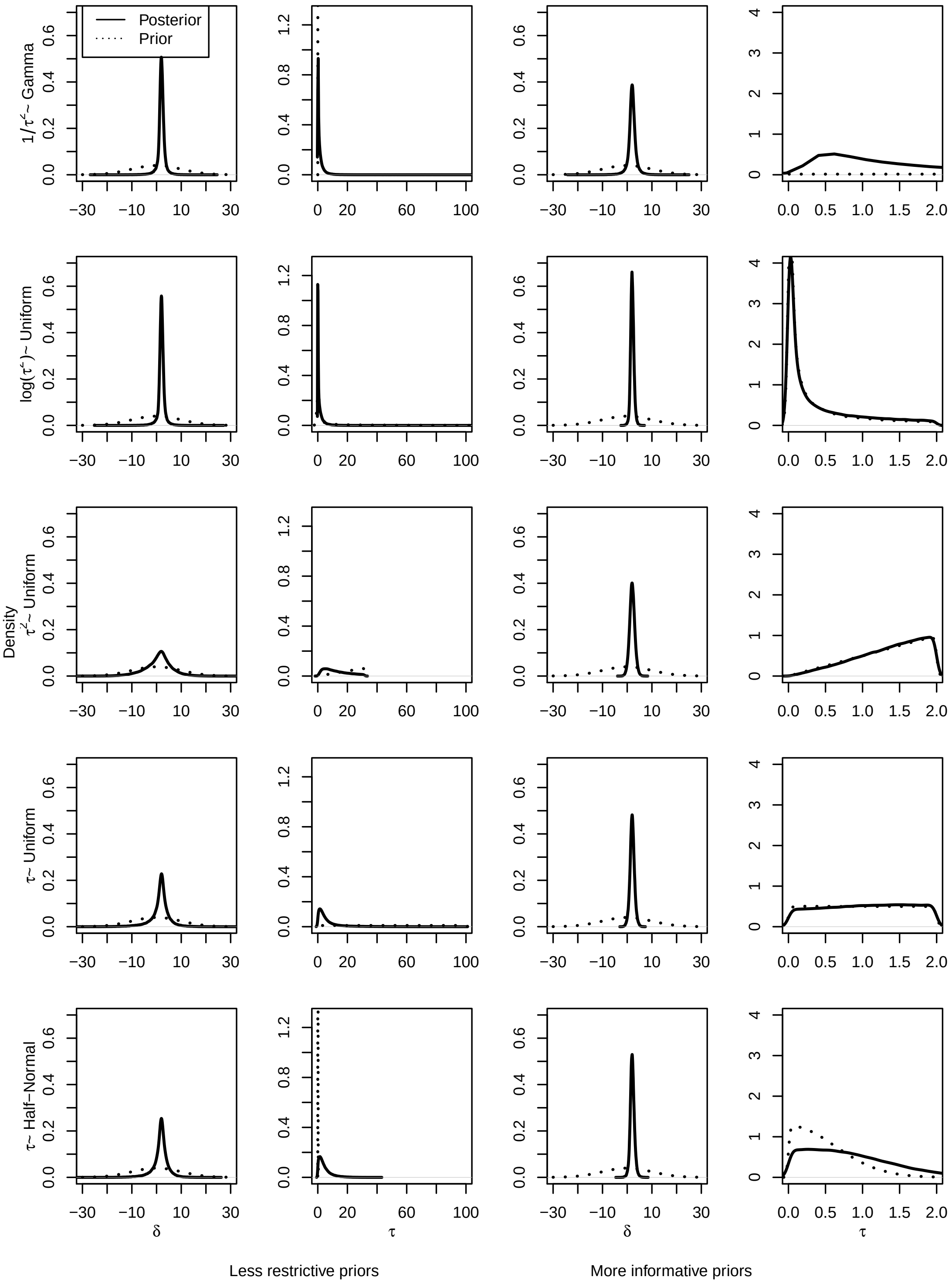

Figure 2: Priors and posterior distributions of the 10 non-empirical heterogeneity priors for the overall treatment effect (δ) and the between-study standard deviation (τ) applied on the Guillain-Barre syndrome example 2.

		Number of studies:	2-4	2-4	2	3	4	2	3	4
P_c	δ	Sample $U(\alpha, \beta)$	*SZ*	*DZ*		*All SZ*			*All DZ*	
0.05	0	(5,10)	89.50	46.27	70.73	52.47	37.86	21.18	9.67	4.36
0.10	0	(5,10)	70.03	21.40	37.06	18.12	8.75	4.54	1	0.19
0.30	0	(5,10)	16.64	1.01	1.55	0.20	0.02	0.02	0	0
0.05	0.50	(5,10)	85.13	37	62.14	42.31	28.42	13.26	4.81	1.72
0.10	0.50	(5,10)	61.45	14.29	27.65	12.17	5.27	2.08	0.27	0.03
0.30	0.50	(5,10)	11.05	0.37	0.75	0.07	0.01	0	0	0
0.05	2	(5,10)	71.56	8.16	47.03	31.97	21.73	0.59	0.05	0.01
0.10	2	(5,10)	46.61	1.31	20.46	9.50	4.39	0.02	0	0
0.30	2	(5,10)	7.93	0	0.63	0.07	0.01	0	0	0
0.05	0	(40,50)	20.77	1.21	2.58	0.36	0.05	0.03	0	0
0.10	0	(40,50)	2.90	0	0.03	0	0	0	0	0
0.30	0	(40,50)	0	0	0	0	0	0	0	0
0.05	0.50	(40,50)	13.40	0.36	1.18	0.12	0.02	0	0	0
0.10	0.50	(40,50)	1.25	0	0.02	0	0	0	0	0
0.30	0.50	(40,50)	0	0	0	0	0	0	0	0
0.05	2	(40,50)	9.87	0	1.03	0.11	0.02	0	0	0
0.10	2	(40,50)	0.91	0	0.02	0	0	0	0	0
0.30	2	(40,50)	0	0	0	0	0	0	0	0

Table 1: Calibration of probability in (%) for trials with a least a single zero arm (SZ), with both zero arms (DZ), all trials with at least one zero arm (All SZ), all trials with both zero arms (All DZ) in a simulated meta-analysis, under combinations of control group event rates P_c, number of patients sample size Uniform(α, β) and treatment effect $\theta = log(OR)$ for 2 to 4 studies.

							Multifocal Neuropathy									
$g(\tau)$	δ	SD_δ	$2.5\%_\delta$	Median δ	$97.5\%_\delta$	δ^*	SD_{δ^*}	$2.5\%^*_\delta$	Median δ^*	$97.5\%^*_\delta$	τ	SD_τ	$2.5\%_\tau$	Median τ	$97.5\%_\tau$	$Pr(\delta < 0)$	$g(\tau)$
$v_\tau \sim G(0.001, 0.001)$	2.64	2.99	-2.97	2.38	9.83	2.64	7.76	-9.51	2.34	16.28	3.09	6.84	0.03	0.83	19.22	0.92	AG
$v_\tau \sim G(0.1, 0.1)$	2.8	3.25	-3.55	2.54	10.37	2.81	8.15	-10.67	2.49	17.58	3.8	6.34	0.27	1.76	19.4	0.9	ag
$log(\tau^2) \sim U(-10, 10)$	2.56	2.72	-2.3	2.34	9.07	2.57	6.78	-7.64	2.3	14.55	2.43	5.71	0.01	0.36	17.18	0.93	AU
$log(\tau^2) \sim U(-10, 1.386)$	2.31	1.19	0.16	2.24	4.86	2.3	1.35	-0.13	2.23	5.18	0.37	0.5	0.01	0.13	1.76	0.98	du
$\tau^2 \sim U(0, 1000)$	2.91	7.1	-11.81	3	16.99	2.96	19.51	-39.07	3.15	43.7	16.3	8.01	2.81	15.89	30.61	0.68	B
$\tau^2 \sim U(0, 4)$	2.54	1.54	-0.33	2.47	5.79	2.54	2.1	-1.53	2.47	6.94	1.35	0.47	0.33	1.43	1.98	0.96	b
$\tau \sim U(0, 100)$	3.27	3.72	-4	2.94	11.68	3.27	6.56	-10.42	2.86	17.87	4.64	2.79	0.23	4.45	9.66	0.86	C
$\tau \sim U(0, 2)$	2.45	1.42	-0.14	2.37	5.49	2.45	1.84	-1.1	2.37	6.42	1.02	0.58	0.05	1.04	1.95	0.97	c
$\tau \sim HN(0, 100)$	3.05	5.7	-9.38	2.91	15.01	3.04	16.01	-31.62	2.87	36.43	10.99	10.13	0.4	8.07	38.03	0.77	DN
$\tau \sim HN(0, 1)$	2.39	1.35	-0.04	2.31	5.3	2.39	1.69	-0.77	2.31	6.05	0.82	0.61	0.03	0.7	2.27	0.97	dn
$s_0/(s_0 + \tau) \sim U(0, 1)$	2.51	1.5	-0.26	2.44	5.69	2.51	2.02	-1.38	2.44	6.75	1.24	0.52	0.28	1.24	2.24	0.96	E
$\tau^2 \sim HN(0, \Phi(0.75)/s_0)$	2.76	2.86	-2.51	2.49	9.53	2.77	5.85	-7.45	2.44	14.58	2.89	4.19	0.05	1.54	13.75	0.92	e

							Guillain-Barre Syndrome									
	δ	SD_δ	$2.5\%_\delta$	Median δ	$97.5\%_\delta$	δ^*	SD_{δ^*}	$2.5\%^*_\delta$	Median δ^*	$97.5\%^*_\delta$	τ	SD_τ	$2.5\%_\tau$	Median τ	$97.5\%_\tau$	$Pr(\delta < 0)$	$g(\tau)$
$v_\tau \sim G(0.001, 0.001)$	-2.61	2.01	-7.13	-2.51	1.31	-2.61	4.65	-11.59	-2.51	5.79	2.22	3.58	0.04	0.96	11.6	0.95	AG
$v_\tau \sim G(0.1, 0.1)$	-2.64	2.26	-7.7	-2.54	1.77	-2.64	5.21	-12.81	-2.54	6.88	2.87	3.91	0.28	1.69	12.58	0.94	ag
$log(\tau^2) \sim U(-10, 10)$	-2.6	1.86	-6.73	-2.51	0.91	-2.58	4.34	-10.7	-2.5	4.86	1.82	3.47	0.01	0.47	10.95	0.96	AU
$log(\tau^2) \sim U(-10, 1.386)$	-2.52	0.73	-4.07	-2.49	-1.14	-2.52	1	-4.64	-2.49	-0.43	0.43	0.54	0.01	0.15	1.82	1	du
$\tau^2 \sim U(0, 1000)$	-2.74	5.69	-14.43	-2.75	9.4	-2.75	15.95	-36.88	-2.79	32.24	12.75	7.7	1.93	11.27	29.5	0.73	B
$\tau^2 \sim U(0, 4)$	-2.52	1.12	-4.82	-2.5	-0.34	-2.52	1.84	-6.29	-2.51	1.2	1.39	0.45	0.38	1.48	1.98	0.99	b
$\tau \sim U(0, 100)$	-2.8	2.81	-9.1	-2.65	2.94	-2.8	5.71	-15.53	-2.63	9.35	4.22	2.61	0.28	3.84	9.49	0.88	C
$\tau \sim U(0, 2)$	-2.52	1	-4.6	-2.5	-0.55	-2.52	1.59	-5.87	-2.5	0.79	1.1	0.57	0.07	1.15	1.96	0.99	c
$\tau \sim HN(0, 100)$	-2.77	4.02	-11.58	-2.65	5.93	-2.77	10.69	-25.08	-2.64	19.58	7.13	6.89	0.36	5.05	25.92	0.83	DN
$\tau \sim HN(0, 1)$	-2.52	0.92	-4.46	-2.49	-0.71	-2.52	1.44	-5.56	-2.5	0.47	0.9	0.63	0.04	0.81	2.34	0.99	dn
$s_0/(s_0 + \tau) \sim U(0, 1)$	-2.52	0.83	-4.23	-2.49	-0.93	-2.52	1.21	-4.99	-2.5	-0.09	0.81	0.33	0.19	0.82	1.44	1	E
$\tau^2 \sim HN(0, \Phi(0.75)/s_0)$	-2.58	1.56	-5.95	-2.51	0.34	-2.59	3.12	-8.79	-2.51	3.15	1.6	2.19	0.02	0.9	7.34	0.97	e

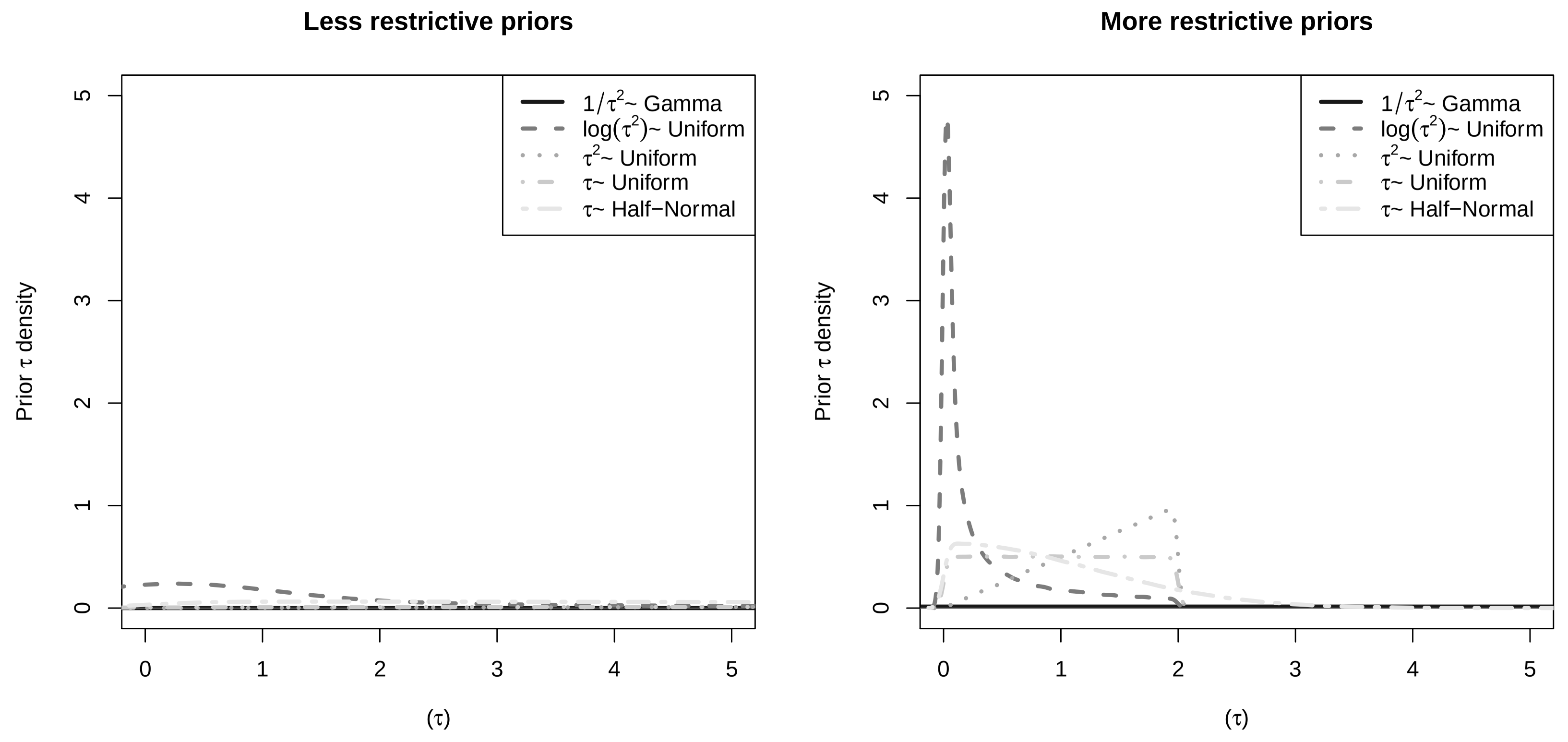

Figure 3: Priors distribution densities of the 10 non-empirical heterogeneity priors for the between-study standard deviation (τ) with fixed axes between less and more informative priors
