# Supplementary material for: Prior distributions for variance parameters in a sparse‐event meta‐analysis of a few small trials
Source: Pharm Stat. 2020 Aug 6;20(1):39–54. doi: 10.1002/pst.2053 (PMC7818503; doi:10.1002/pst.2053)
Supplement: Supplementary file 2 — Data S2. Simulation figures. [file PST-20-39-s002.pdf]

Supplementary material SII - Simulation figures

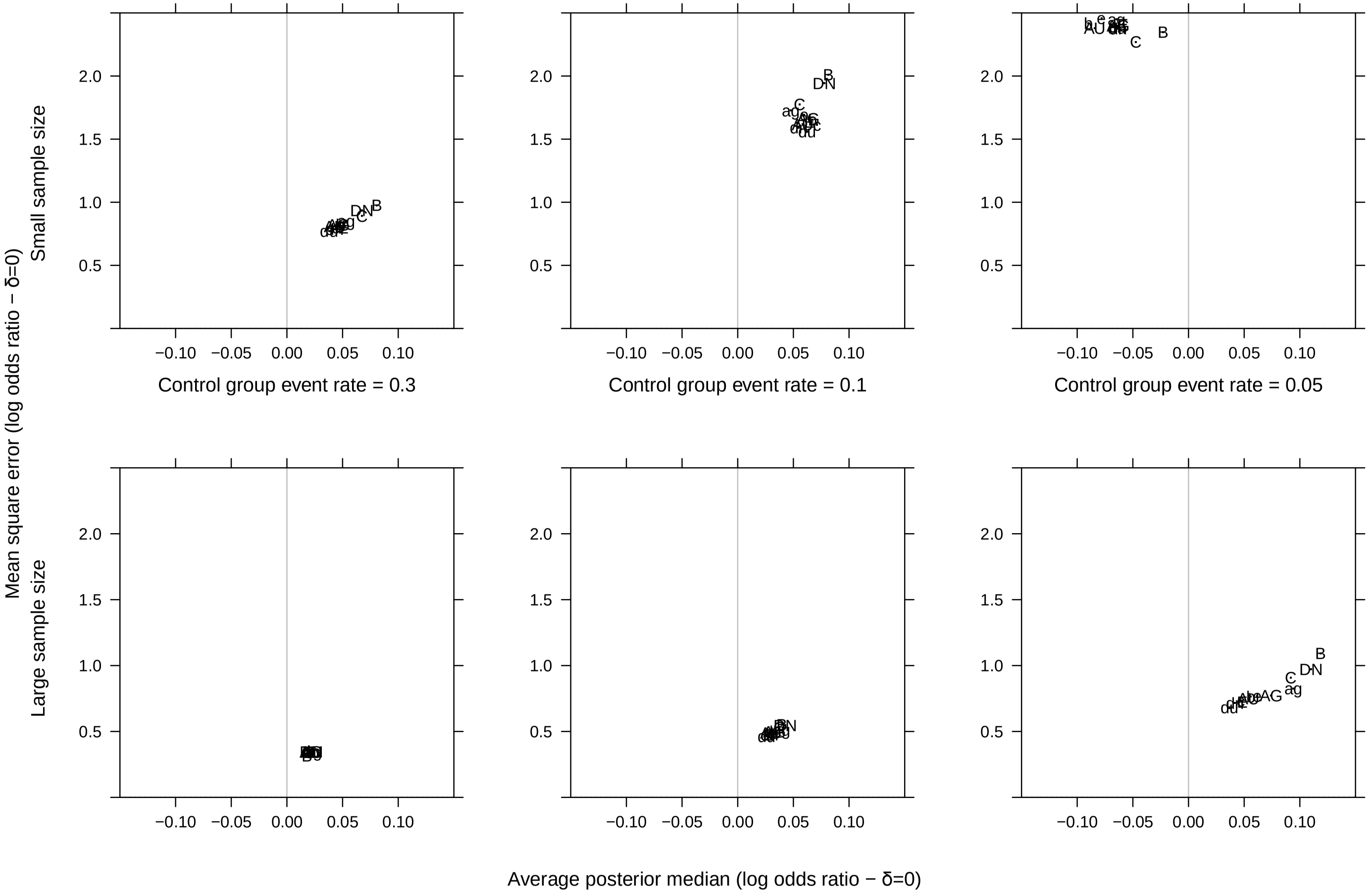

Figure 1: Scatter plot of average posterior median overall effect (log odds ratio) against the mean square error of δ for all simulated scenarios (Overall effect: $\delta = 0$, between-study standard deviation: $\tau \in \{0.01, 0.5, 1\}$, number of trials: $k \in \{2, 4, 6\}$) of a meta-analysis with control group event rate: $\pi_c \in \{0.05, 0.1, 0.3\}$ with small sample size trials ($n_{ij} \sim Uniform(5, 10)$) or large sample sized trials ($n_{ij} \sim Uniform(40, 50)$). (AU, Au) - *Gamma* on v_τ, (AU, du) - *Uniform* on $log(\tau^2)$, (B, b) - *Uniform* on τ^2, (C, c) - *Uniform* on τ, (DN , dn) - *Half-normal* on τ, (e) *Half-normal* on τ^2, (E) - *DuMouchel* prior. (AG, AU, B, C, DN) are less restrictive priors on τ and (ag, dn, b, c, dn) are more informative priors on τ.

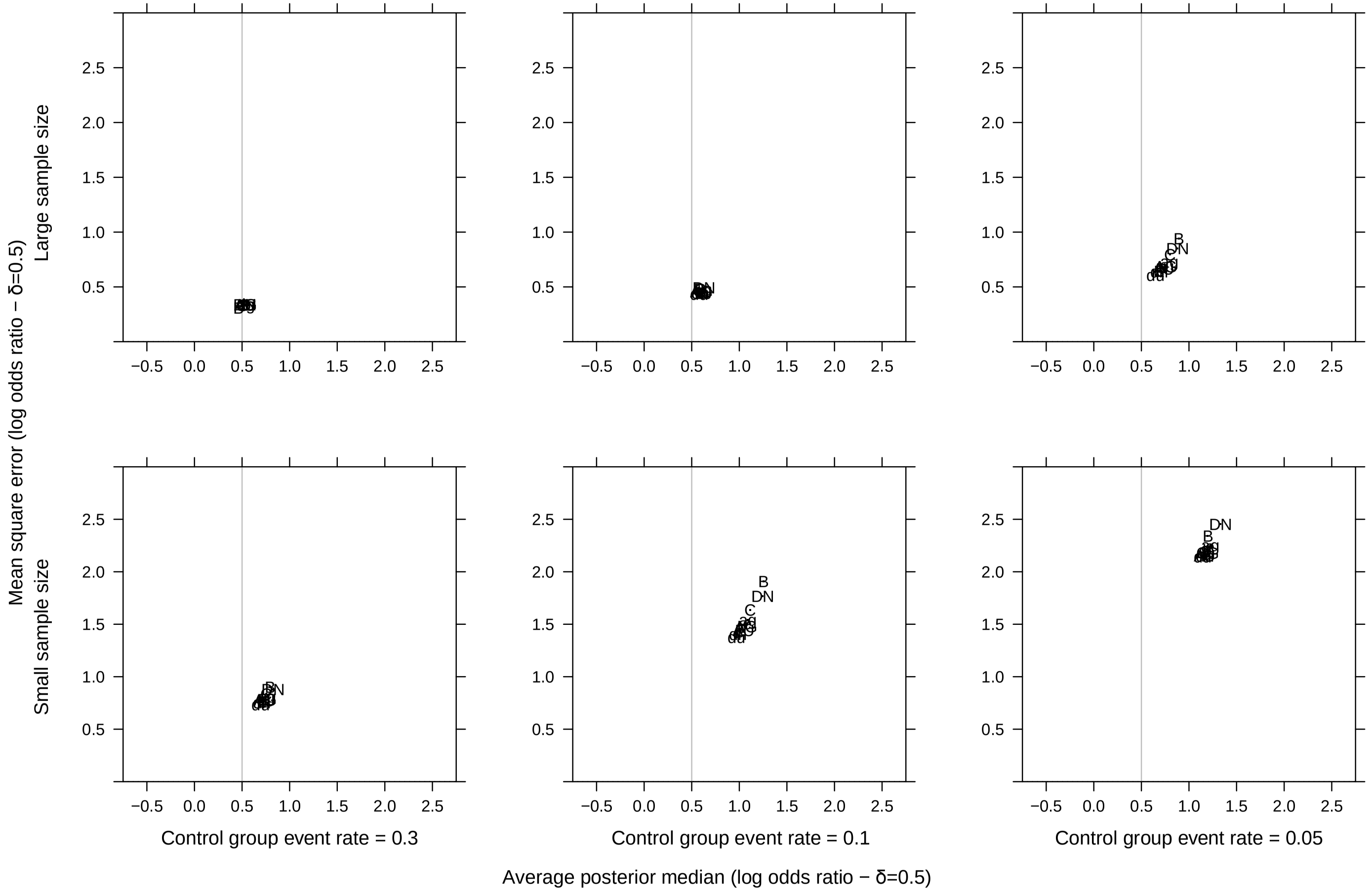

Figure 2: Scatter plot of average posterior median overall effect (log odds ratio) against the mean square error of δ for all simulated scenarios (Overall effect: $\delta = 0.5$, between-study standard deviation: $\tau \in \{0.01, 0.5, 1\}$, number of trials: $k \in \{2, 4, 6\}$) of a meta-analysis with control group event rate: $\pi_c \in \{0.05, 0.1, 0.3\}$ with small sample size trials ($n_{ij} \sim Uniform(5, 10)$) or large sample sized trials ($n_{ij} \sim Uniform(40, 50)$). (AU, Au) - *Gamma* on v_τ, (AU, du) - *Uniform* on $log(\tau^2)$, (B, b) - *Uniform* on τ^2, (C, c) - *Uniform* on τ, (DN , dn) - *Half-normal* on τ, (e) *Half-normal* on τ^2, (E) - *DuMouchel* prior. (AG, AU, B, C, DN) are less restrictive priors on τ and (ag, dn, b, c, dn) are more informative priors on τ.

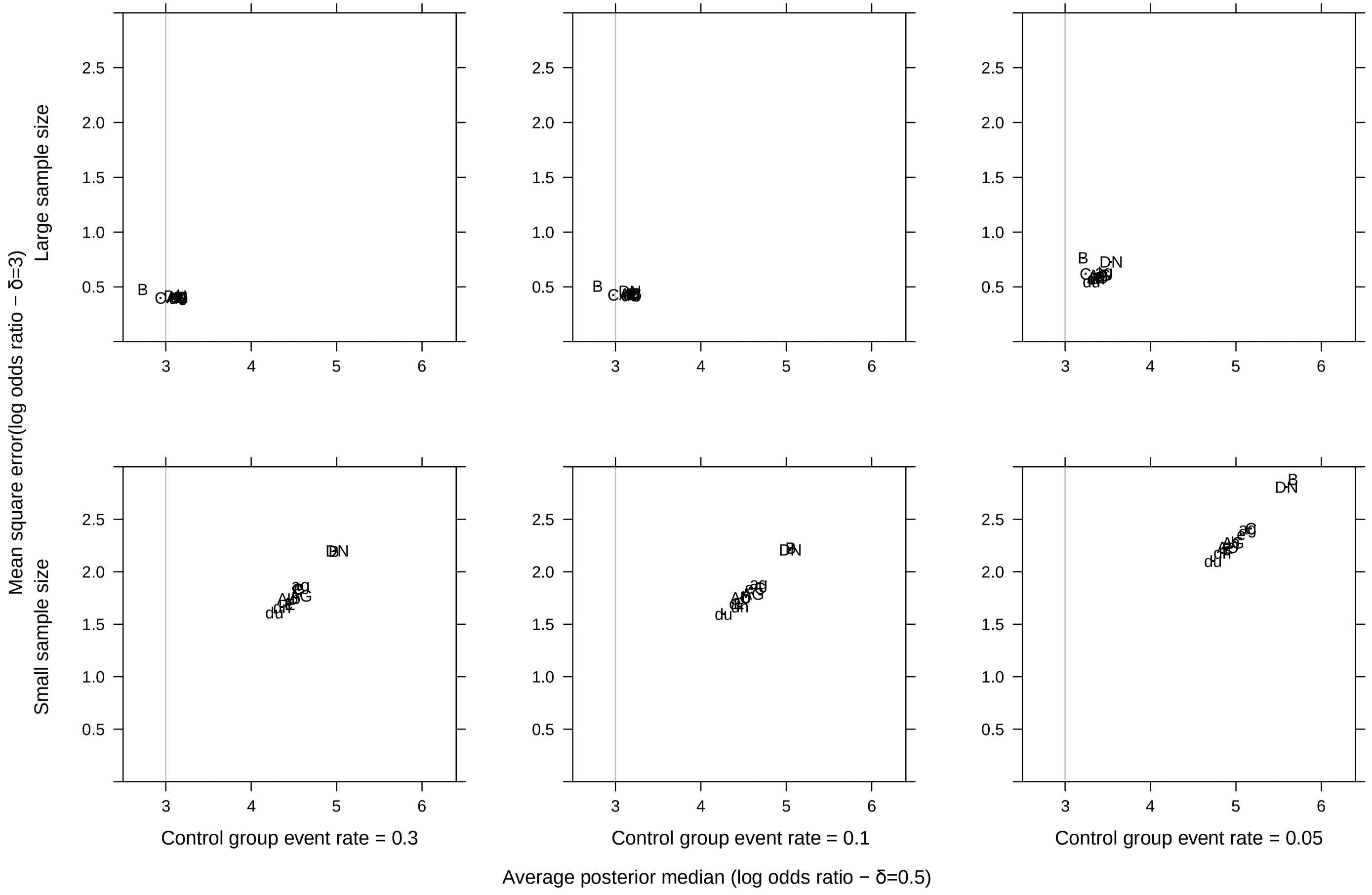

Figure 3: Scatter plot of average posterior median overall effect (log odds ratio) against the mean square error of δ for all simulated scenarios (Overall effect: $\delta = 3$, between-study standard deviation: $\tau \in \{0.01, 0.5, 1\}$, number of trials: $k \in \{2, 4, 6\}$) of a meta-analysis with control group event rate: $\pi_c \in \{0.05, 0.1, 0.3\}$ with small sample size trials ($n_{ij} \sim Uniform(5, 10)$) or large sample sized trials ($n_{ij} \sim Uniform(40, 50)$). (AU, Au) - $Gamma$ on v_τ, (AU, du) - $Uniform$ on $log(\tau^2)$, (B, b) - $Uniform$ on τ^2, (C, c) - $Uniform$ on τ, (DN , dn) - $Half$-$normal$ on τ, (e) $Half$-$normal$ on τ^2, (E) - $DuMouchel$ prior. (AG, AU, B, C, DN) are less restrictive priors on τ and (ag, dn, b, c, dn) are more informative priors on τ.

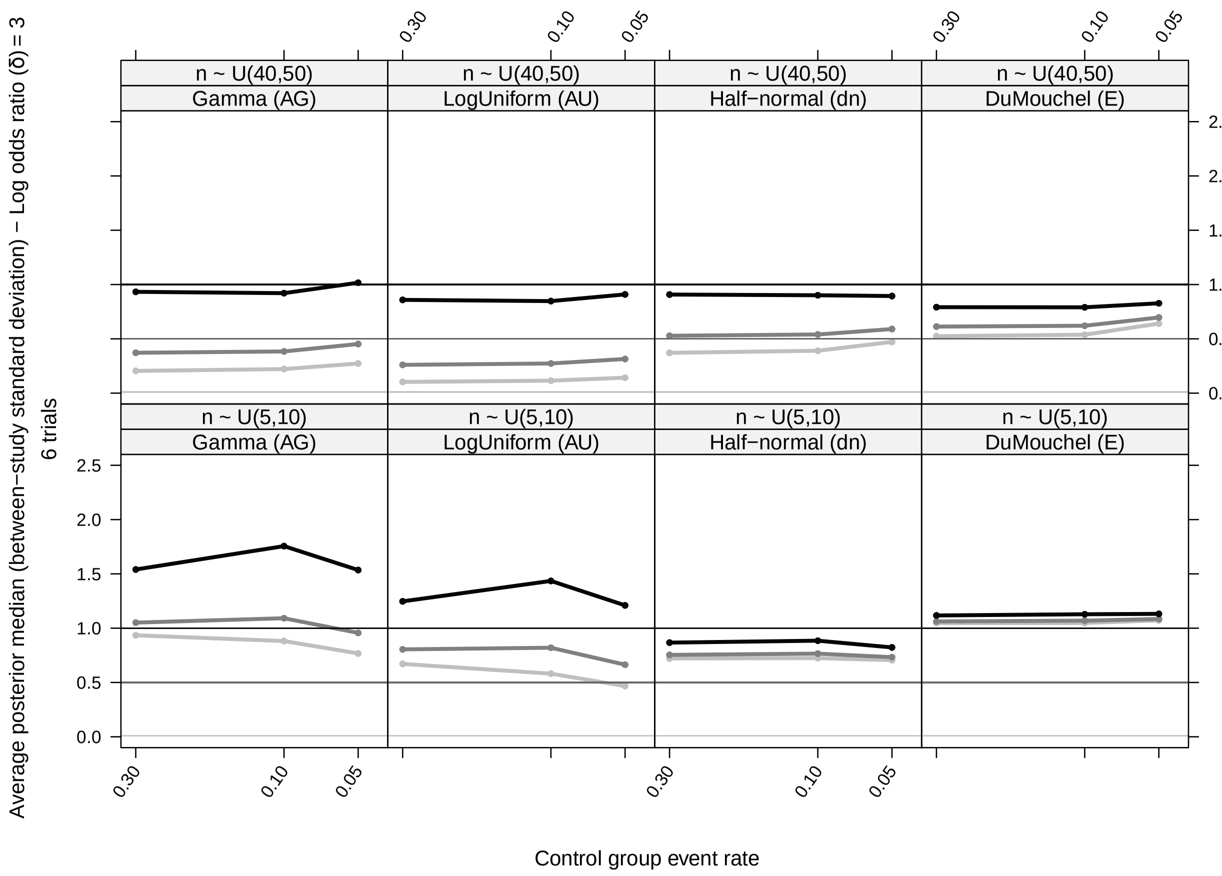

Figure 4: Average posterior median line plots of the between-study standard deviation (τ) on different control group event rate levels for a large true overall effect ($\delta = 3$) and small sample size trials ($n_{ij} \sim Uniform(5, 10)$) or large sample sized trials ($n_{ij} \sim Uniform(40, 50)$). The grey lines represent 3 levels of heterogeneity, namely, light grey: $\tau = 0.01$, grey: $\tau = 0.5$, dark grey: $\tau = 1$ (AG): $Gamma(0.001, 0.001)$ on v_τ, (AU): $Uniform(-10, 10)$ on $log(\tau^2)$, (dn): $Half\text{-}normal(0, 1)$ on τ, (E): $DuMouchel$ prior.

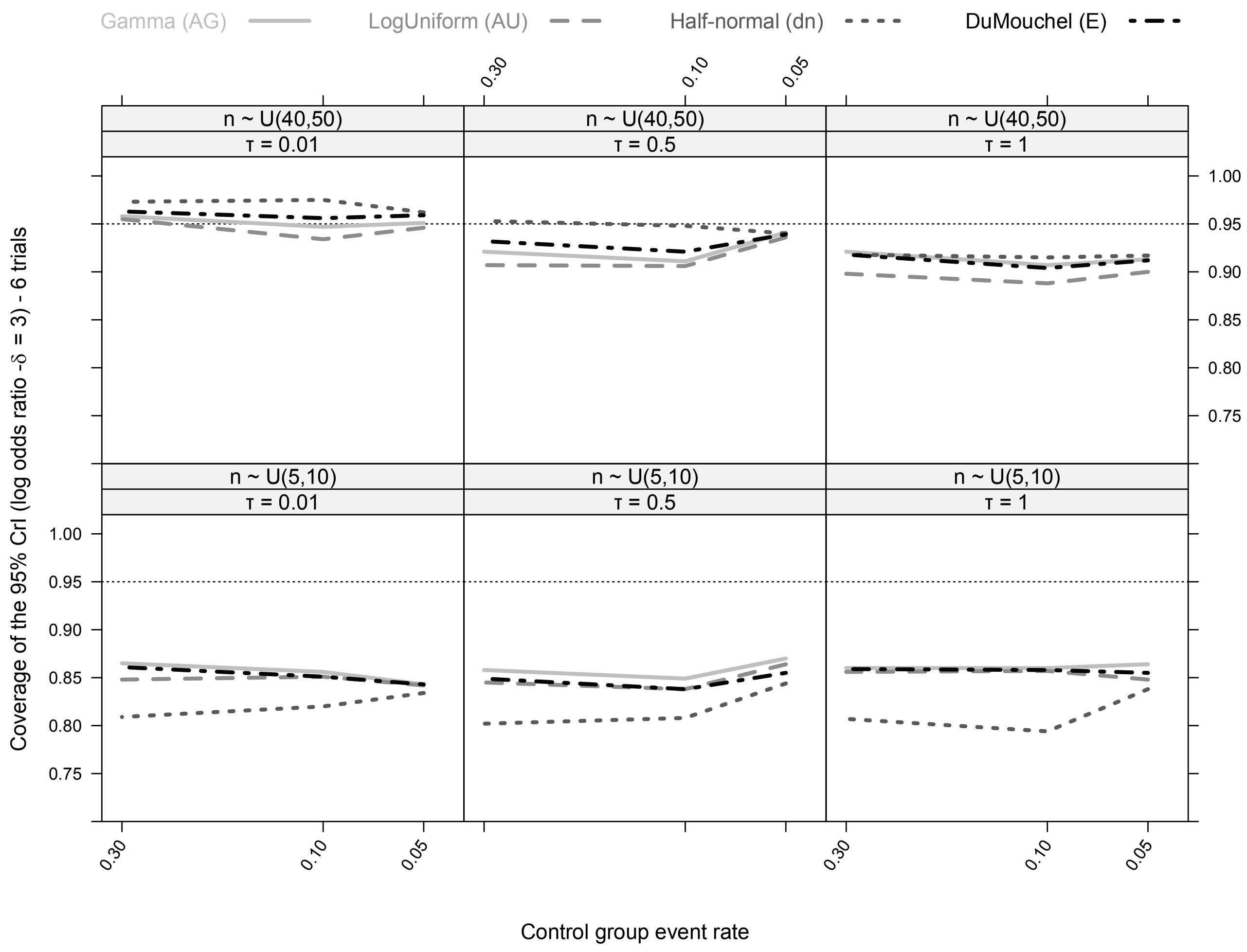

Figure 5: Coverage of the 95% CrI line plots of the overall effect (log odds ratio) on different control group event rate levels for a large true overall effect ($\delta = 3$), three values of $\tau \in \{0.01, 0.5, 1\}$ and small sample size trials ($n_{ij} \sim Uniform(5, 10)$) or large sample sized trials ($n_{ij} \sim Uniform(40, 50)$). (AG): $Gamma(0.001, 0.001)$ on v_τ, (AU): $Uniform(-10, 10)$ on $log(\tau^2)$, (dn): $Half\text{-}normal(0, 1)$ on τ, (E): $DuMouchel$ prior.

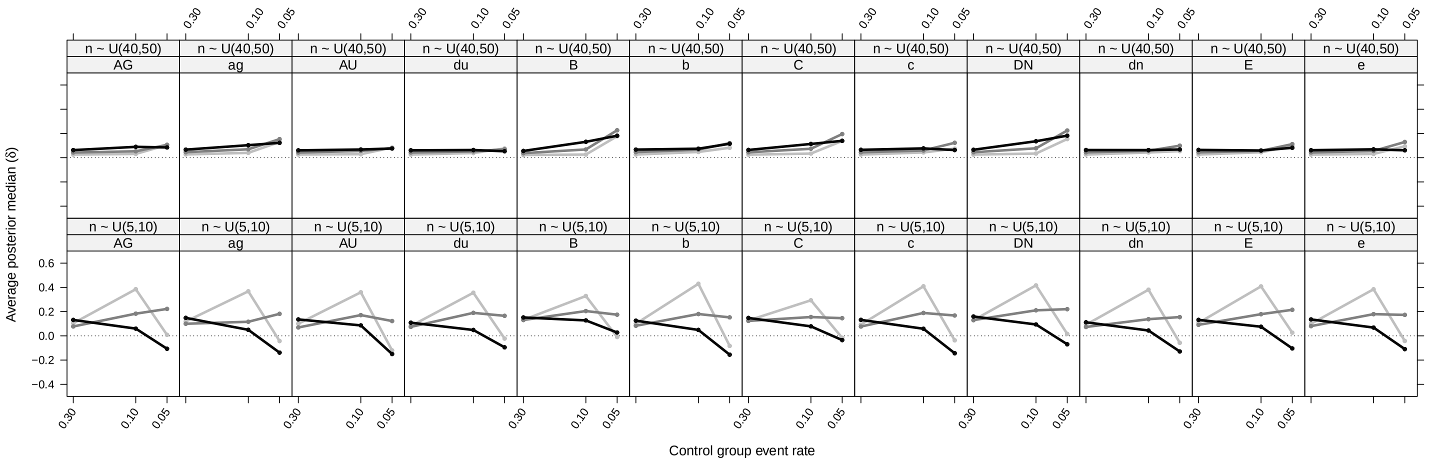

Figure 6: Line plots representing mean posterior median of the overall treatment effect on different levels of average risk rate for a meta-analysis of 2 trials under the null hypothesis ($\delta = 0$). The grey lines represent 3 levels of heterogeneity, namely, light grey: $\tau_r = 0.01$, grey: $\tau_r = 1$, dark grey: $\tau_r = 2$ (AG,ag) : Gamma on τ^{-2}, (AU,du) : Uniform on $log(\tau^2)$, (C,c) : Uniform on τ^2, (D,d) : Uniform on τ, (DN,dn) : Half-normal on τ, (e) Half-normal on τ^2, (E) : DuMouchel prior. (ag, du, b, c, dn) are either less restrictive and (AG, AU, B, C, DN) are either more informative. A similar behaviour was observed for a meta-analysis of 4 and 6 trials.

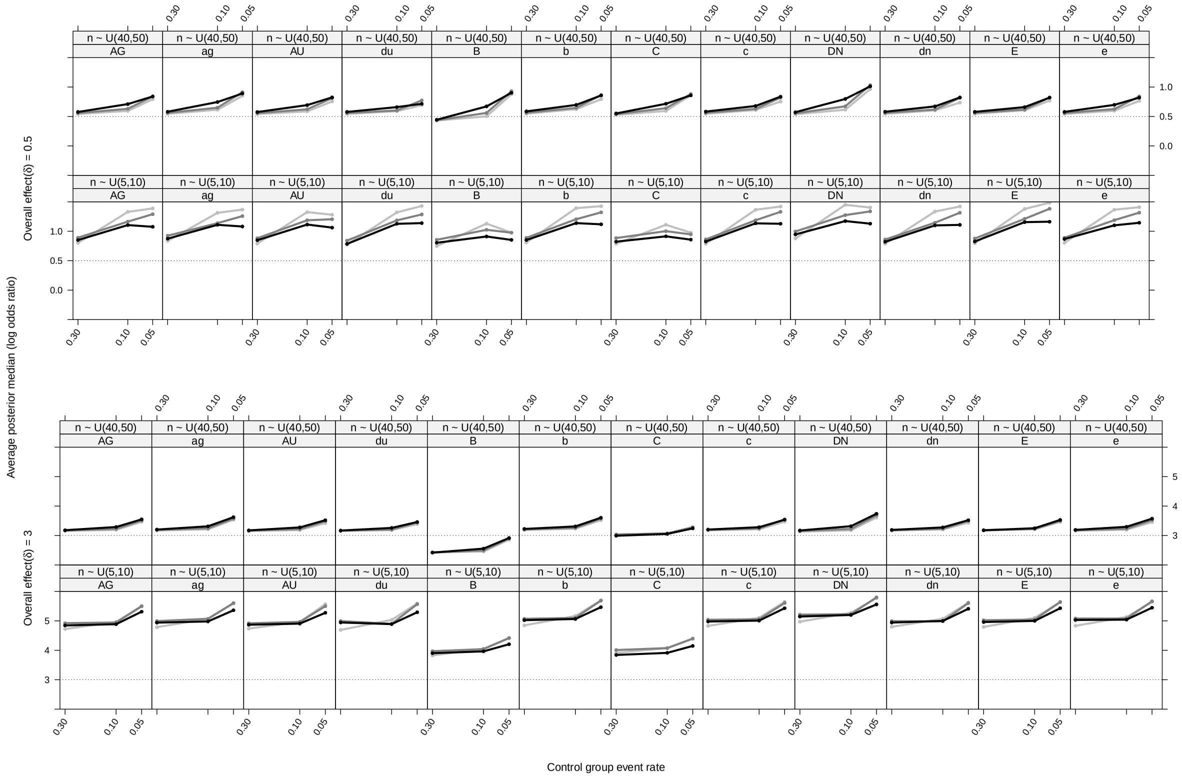

Figure 7: Line plots representing mean posterior median of the overall treatment effect on different levels of average risk rate for a meta-analysis of 2 trials. The grey lines represent 3 levels of heterogeneity, namely, light grey: $\tau_r = 0.01$, grey: $\tau_r = 1$, dark grey: $\tau_r = 2$ (AG,ag) : Gamma on τ^{-2}, (AU,du) : Uniform on $log(\tau^2)$, (C,c) : Uniform on τ^2, (D,d) : Uniform on τ, (DN,dn) : Half-normal on τ, (e) Half-normal on τ^2, (E) : DuMouchel prior. (ag, du, b, c, dn) are either less restrictive and (AG, AU, B, C, DN) are either more informative.

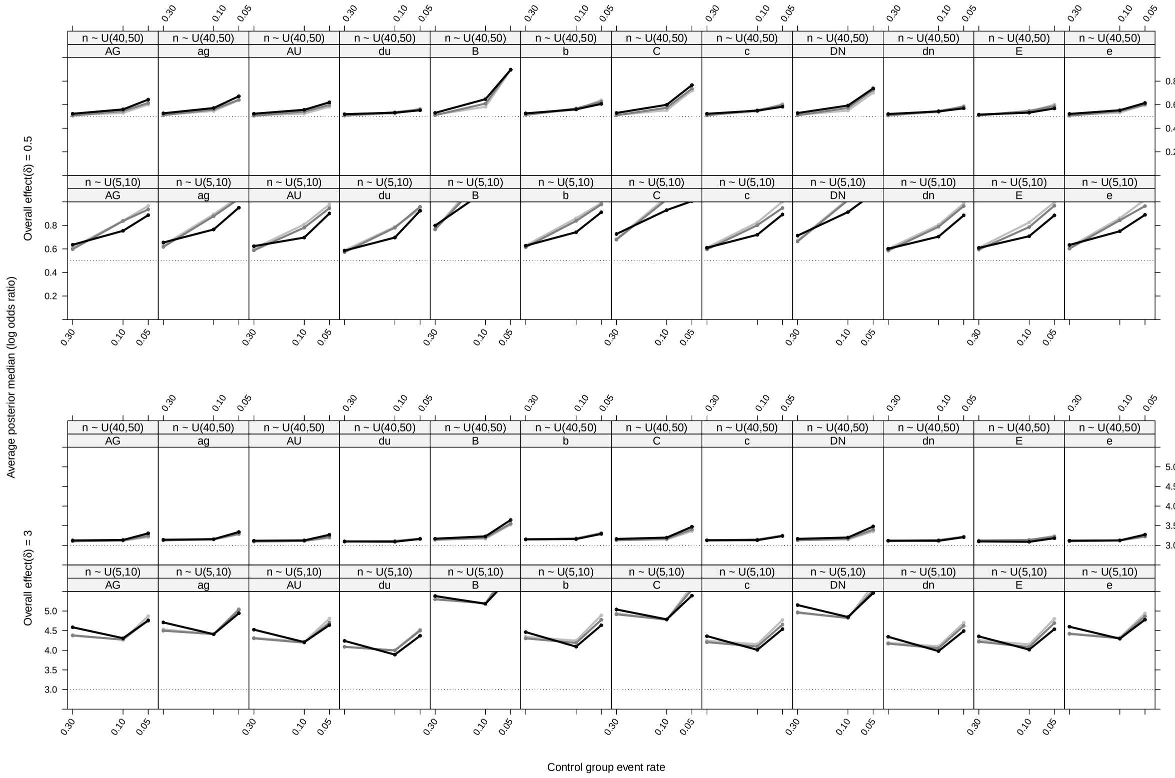

Figure 8: Line plots representing mean posterior median of the overall treatment effect on different levels of average risk rate for a meta-analysis of 4 trials. The grey lines represent 3 levels of heterogeneity, namely, light grey: $\tau_r = 0.01$, grey: $\tau_r = 1$, dark grey: $\tau_r = 2$ (AG,ag) : Gamma on τ^{-2}, (AU,du) : Uniform on $log(\tau^2)$, (C,c) : Uniform on τ^2, (D,d) : Uniform on τ, (DN,dn) : Half-normal on τ, (e) Half-normal on τ^2, (E) : DuMouchel prior. (ag, du, b, c, dn) are either less restrictive and (AG, AU, B, C, DN) are either more informative.

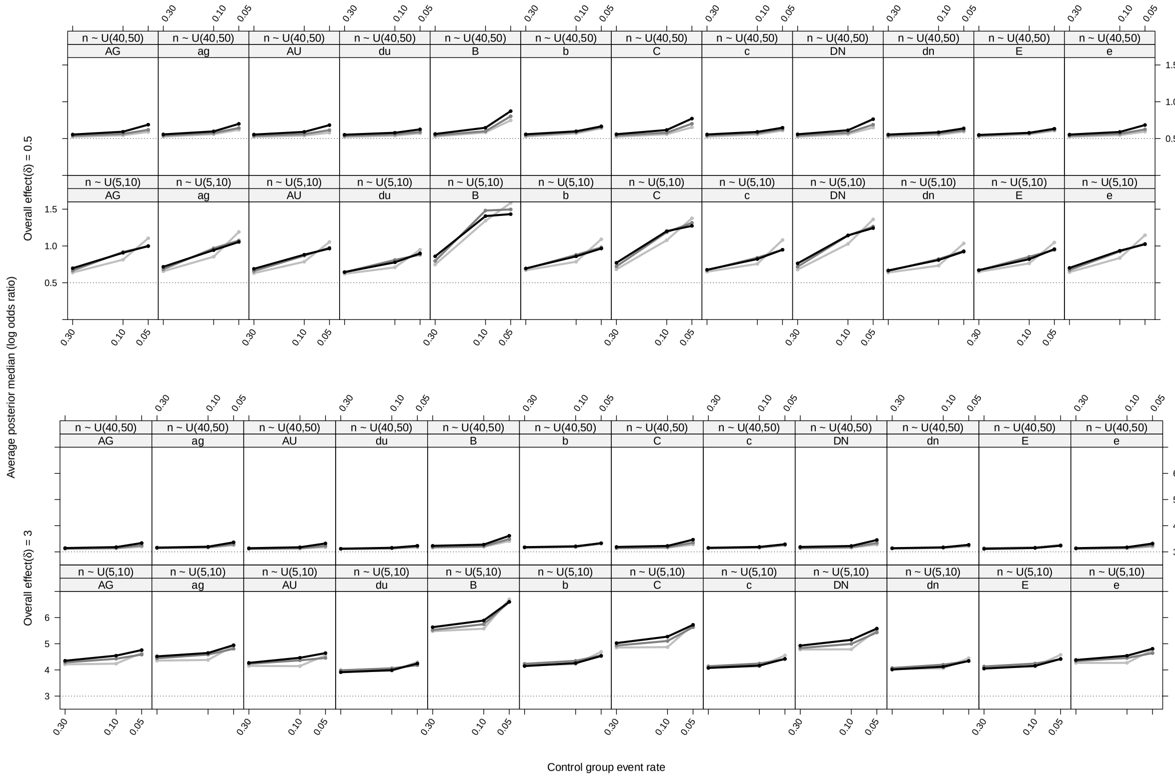

Figure 9: Line plots representing mean posterior median of the overall treatment effect on different levels of average risk rate for a meta-analysis of 6 trials. The grey lines represent 3 levels of heterogeneity, namely, light grey: $\tau_r = 0.01$, grey: $\tau_r = 1$, dark grey: $\tau_r = 2$ (AG,ag) : Gamma on τ^{-2}, (AU,du) : Uniform on $log(\tau^2)$, (C,c) : Uniform on τ^2, (D,d) : Uniform on τ, (DN,dn) : Half-normal on τ, (e) Half-normal on τ^2, (E) : DuMouchel prior. (ag, du, b, c, dn) are either less restrictive and (AG, AU, B, C, DN) are either more informative.

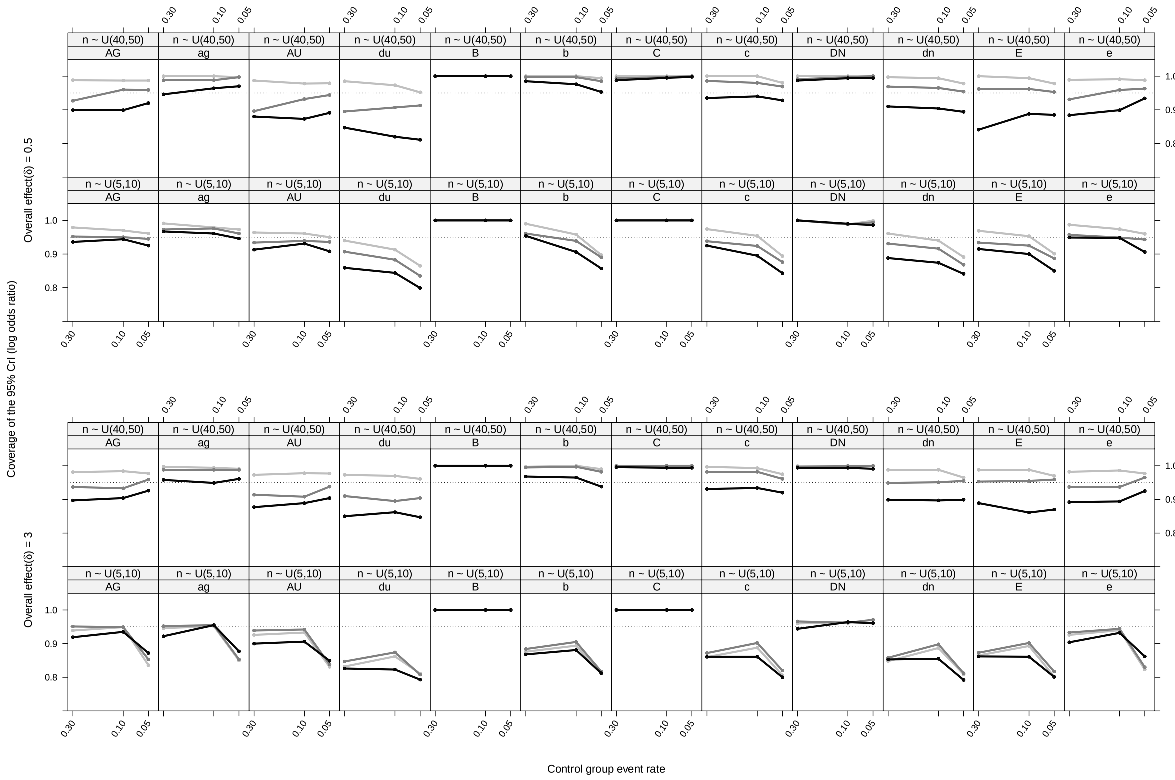

Figure 10: Line plots representing coverage probability of the overall treatment effect on different levels of average risk rate for a meta-analysis of 4 trials. The grey lines represent 3 levels of heterogeneity, namely, light grey: $\tau_r = 0.01$, grey: $\tau_r = 1$, dark grey: $\tau_r = 2$ (AG,ag) : Gamma on τ^{-2}, (AU,du) : Uniform on $log(\tau^2)$, (C,c) : Uniform on τ^2, (D,d) : Uniform on τ, (DN,dn) : Half-normal on τ, (e) Half-normal on τ^2, (E) : DuMouchel prior. (ag, du, b, c, dn) are either less restrictive and (AG, AU, B, C, DN) are either more informative.

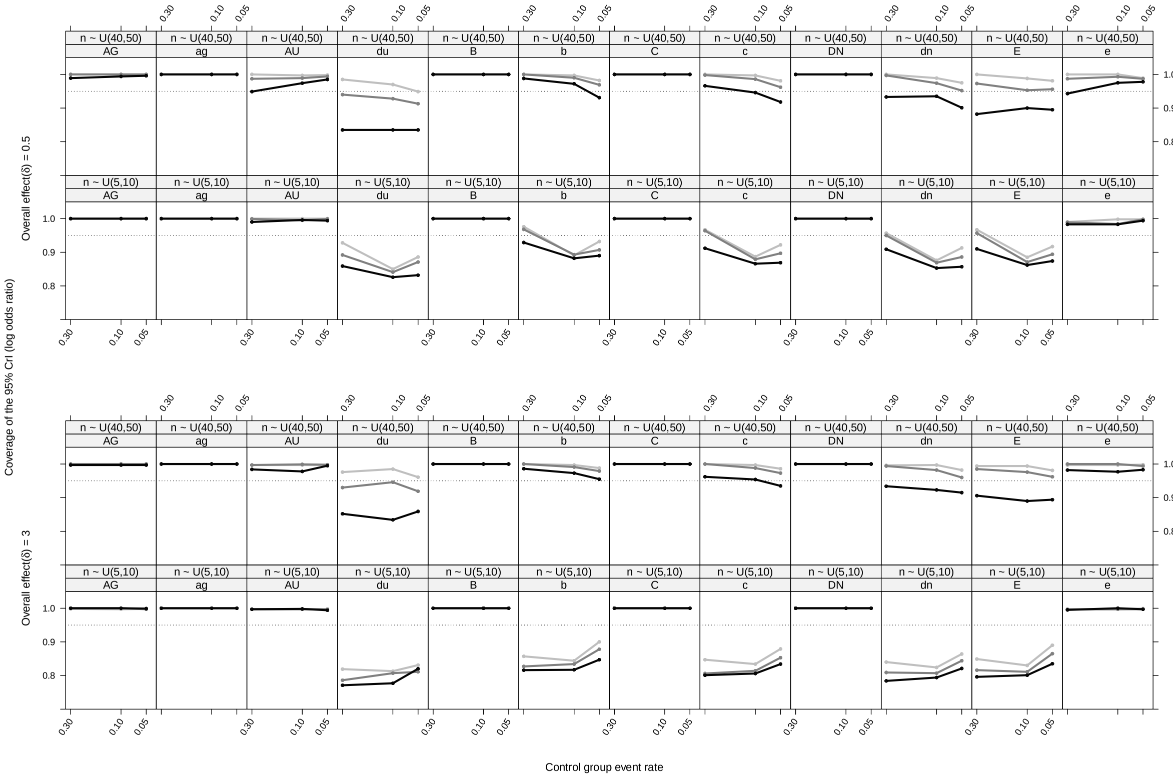

Figure 11: Line plots representing coverage probability of the overall treatment effect on different levels of average risk rate for a meta-analysis of 2 trials. The grey lines represent 3 levels of heterogeneity, namely, light grey: $\tau_r = 0.01$, grey: $\tau_r = 1$, dark grey: $\tau_r = 2$ (AG,ag) : Gamma on τ^{-2}, (AU,du) : Uniform on $log(\tau^2)$, (C,c) : Uniform on τ^2, (D,d) : Uniform on τ, (DN,dn) : Half-normal on τ, (e) Half-normal on τ^2, (E) : DuMouchel prior. (ag, du, b, c, dn) are either less restrictive and (AG, AU, B, C, DN) are either more informative.

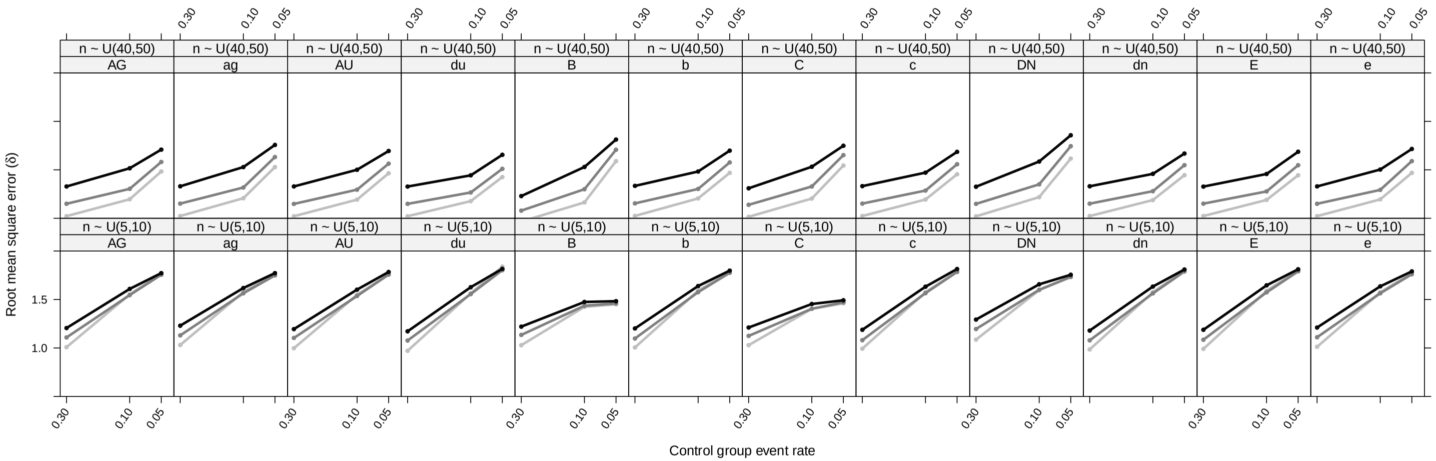

Figure 12: Line plots representing root mean square error of the overall treatment effect on different levels of average risk rate for a meta-analysis of 2 trials under the null hypothesis $\delta = 0$. The grey lines represent 3 levels of heterogeneity, namely, light grey: $\tau_r = 0.01$, grey: $\tau_r = 1$, dark grey: $\tau_r = 2$ (AG,ag) : Gamma on τ^{-2}, (AU,du) : Uniform on $log(\tau^2)$, (C,c) : Uniform on τ^2, (D,d) : Uniform on τ, (DN,dn) : Half-normal on τ, (e) Half-normal on τ^2, (E) : DuMouchel prior. (ag, du, b, c, dn) are either less restrictive and (AG, AU, B, C, DN) are either more informative. A similar behaviour was observed for a meta-analysis of 4 and 6 trials.

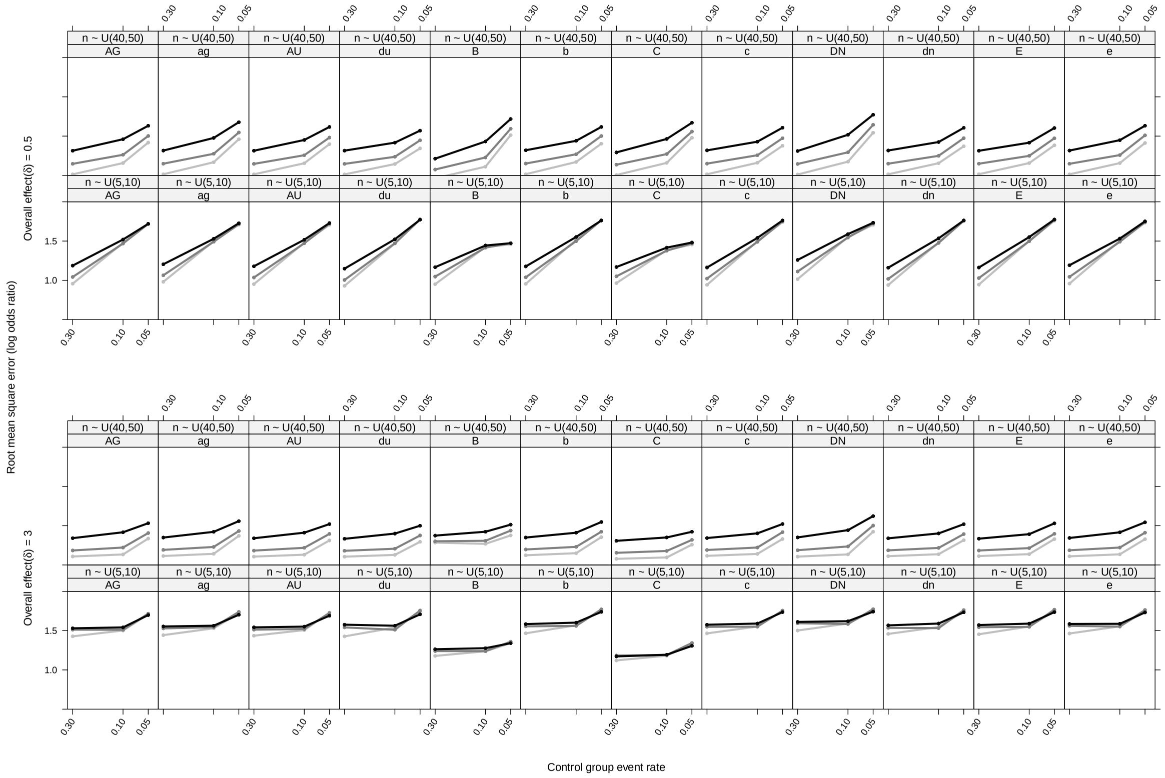

Figure 13: Line plots representing root mean square error of the overall treatment effect on different levels of average risk rate for a meta-analysis of 2 trials. The grey lines represent 3 levels of heterogeneity, namely, light grey: $\tau_r = 0.01$, grey: $\tau_r = 1$, dark grey: $\tau_r = 2$ (AG,ag) : Gamma on τ^{-2}, (AU,du) : Uniform on $log(\tau^2)$, (C,c) : Uniform on τ^2, (D,d) : Uniform on τ, (DN,dn) : Half-normal on τ, (e) Half-normal on τ^2, (E) : DuMouchel prior. (ag, du, b, c, dn) are either less restrictive and (AG, AU, B, C, DN) are either more informative.

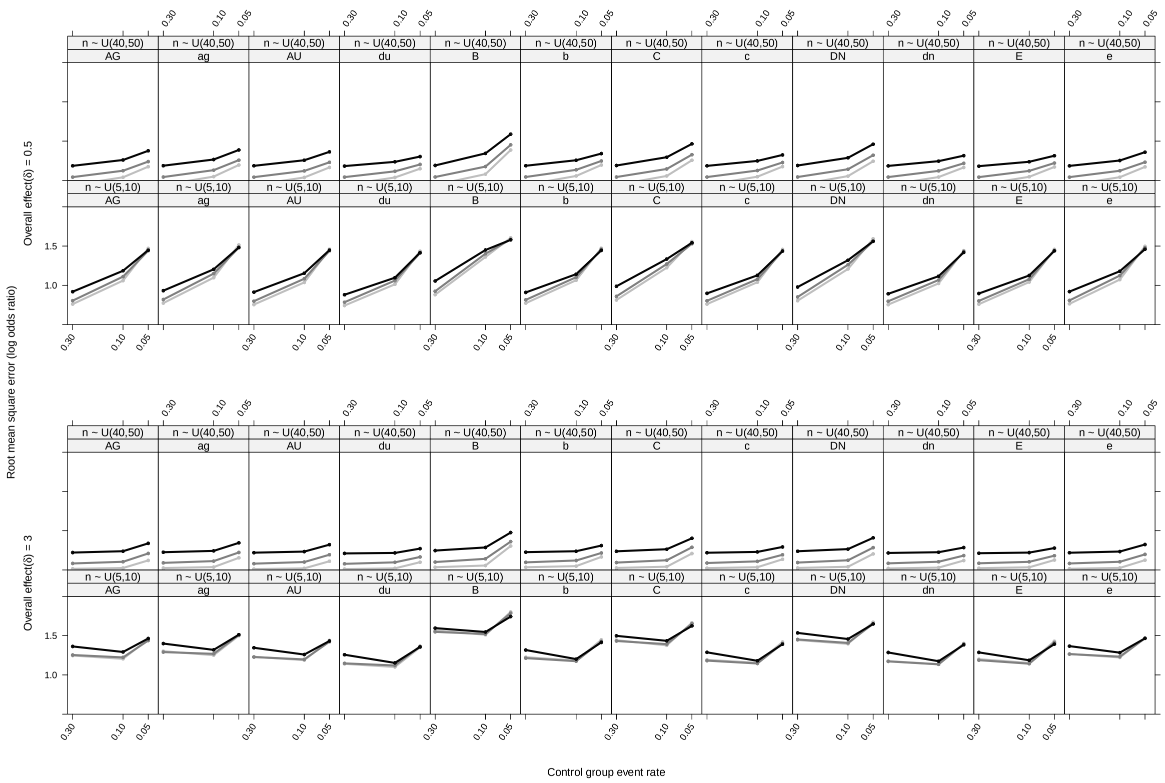

Figure 14: Line plots representing root mean square error of the overall treatment effect on different levels of average risk rate for a meta-analysis of 4 trials. The grey lines represent 3 levels of heterogeneity, namely, light grey: $\tau_r = 0.01$, grey: $\tau_r = 1$, dark grey: $\tau_r = 2$ (AG,ag) : Gamma on τ^{-2}, (AU,du) : Uniform on $log(\tau^2)$, (C,c) : Uniform on τ^2, (D,d) : Uniform on τ, (DN,dn) : Half-normal on τ, (e) Half-normal on τ^2, (E) : DuMouchel prior. (ag, du, b, c, dn) are either less restrictive and (AG, AU, B, C, DN) are either more informative.

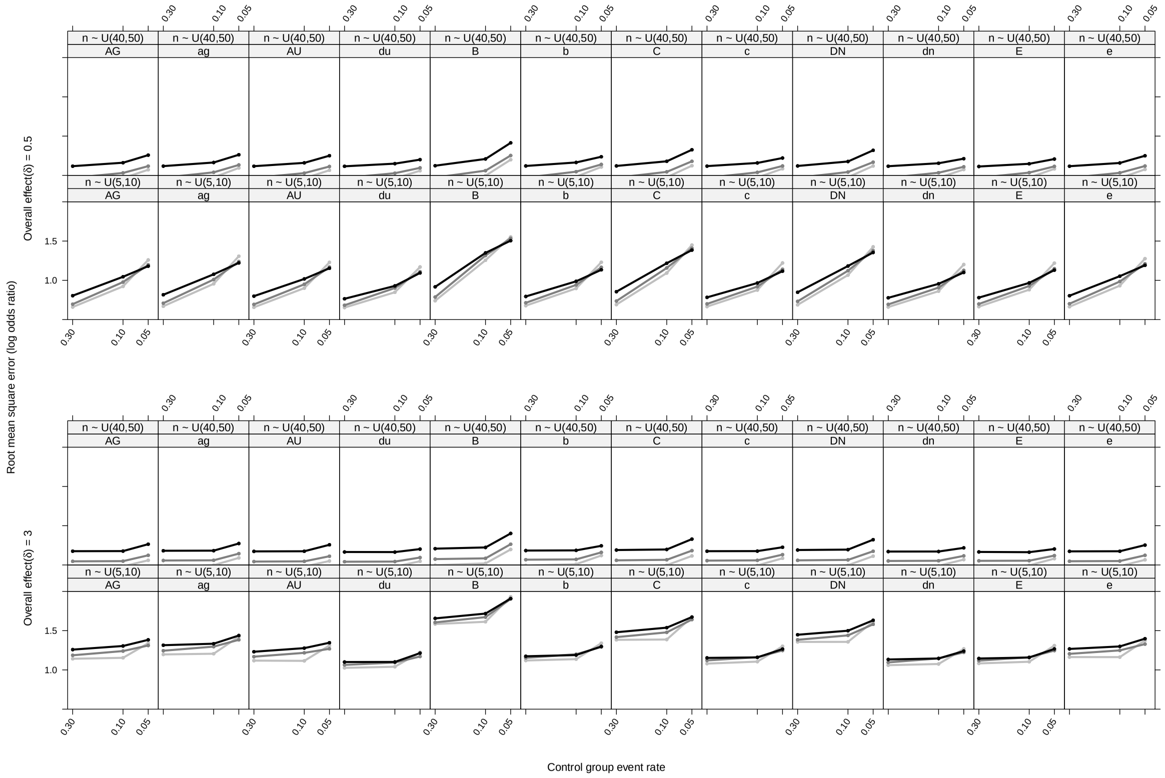

Figure 15: Line plots representing root mean square error of the overall treatment effect on different levels of average risk rate for a meta-analysis of 6 trials. The grey lines represent 3 levels of heterogeneity, namely, light grey: $\tau_r = 0.01$, grey: $\tau_r = 1$, dark grey: $\tau_r = 2$ (AG,ag) : Gamma on τ^{-2}, (AU,du) : Uniform on $log(\tau^2)$, (C,c) : Uniform on τ^2, (D,d) : Uniform on τ, (DN,dn) : Half-normal on τ, (e) Half-normal on τ^2, (E) : DuMouchel prior. (ag, du, b, c, dn) are either less restrictive and (AG, AU, B, C, DN) are either more informative.
