# Supplementary material for: Prior distributions for variance parameters in a sparse‐event meta‐analysis of a few small trials
Source: Pharm Stat. 2020 Aug 6;20(1):39–54. doi: 10.1002/pst.2053 (PMC7818503; doi:10.1002/pst.2053)
Supplement: Supplementary file 3 — Data S3. Diagnostics. [file PST-20-39-s003.pdf]

Supplementary material SIII - Diagnostics

In this section we comment on the behaviour of the Markov Chain Monte Carlo (MCMC) chains for four specific examples under three selected priors of heterogeneity: AG, AU, dn and E under the most possibly problematic scenarios of 50-75% percent single-zero studies.

We visit the example of Multifocal motor neuropathy and we observed our model convergence in Figure 1. In Figure 2, we match our simulation study MCMC iteration number (34,500) and we observe the convergence of a fictional meta-analysis of three small studies with at least three no observed event in either arm.

In Figure 2 we observe that only the prior AG has convergence difficulties regarding the overall effect (δ) and the predictive overall effect (δ^*). However, it samples unrealistically large values for heterogeneity and results in added uncertainty in the interval posterior estimation of δ and δ^*. The other priors AU, E and especially dn show good mixing for τ but have difficulty exploring the tails of their posterior distribution of δ .

In a similar fashion in Figure 1, the less informative prior for the heterogeneity results in less autocorrelation. We accounted for non-convergence issues as we fitted every model via three parallel chains and we accounted for autocorrelation difficulties by applying a thinning interval of 5 MCMC samples.

Finally, in order to investigative the impact of different priors on the overall effect we replicated scenarios for : (1) $\delta \sim U(-10, 10)$, (2) $\delta \sim t(100, df)$, (3) $\delta \sim N(0, 100)I[-10, 10]$. Similar behaviours were observed in our examples. Applying a more restrictive prior on $\delta \sim N(0, 10)$ restricts the posterior point estimate of δ and produces less extreme values but at the same time heavily impacts the posterior credible intervals.

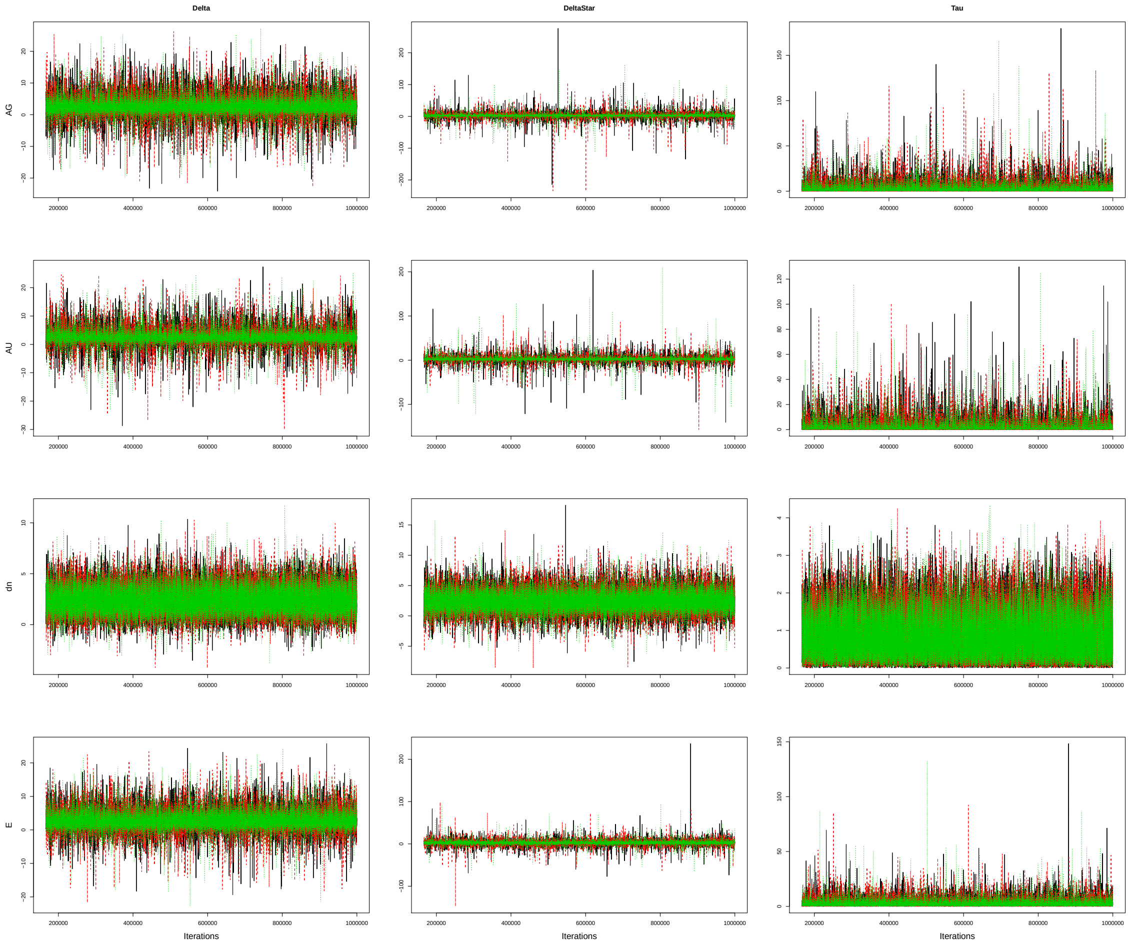

Figure 1: Diagnostic traceplots for δ, δ^* and τ for a selection of priors for the Multifocal Motor Neuropathy example. Traceplots are based on 850,000 iterations, 3 chains, 150,000 burn-in and a thinning of 35 iterations

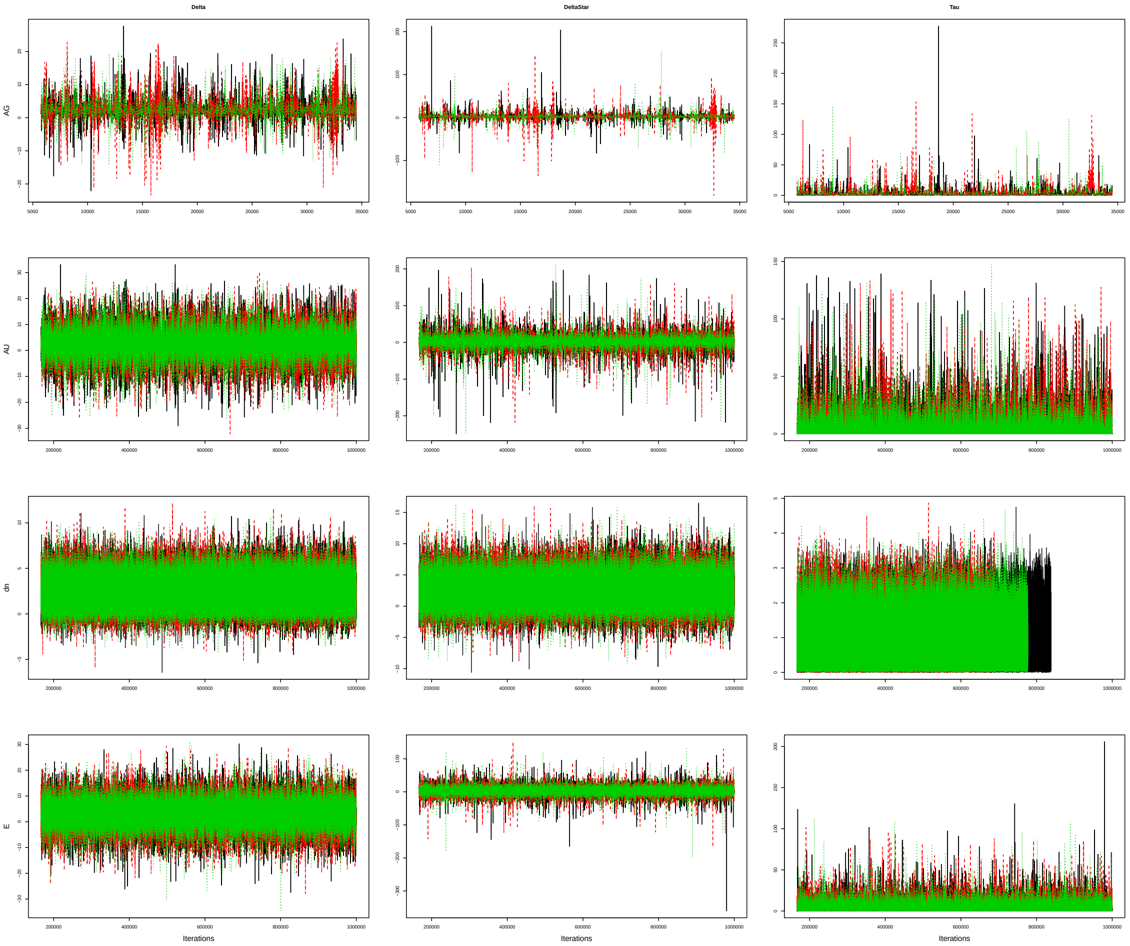

Figure 2: Diagnostic traceplots for δ, δ^* and τ for a selection of priors for the Multifocal Motor Neuropathy example. Traceplots are based on 30,000 iterations, 3 chains, 4,500 burn-in and a thinning of 5 iterations
